# Targeting of a novel interplay between MET tyrosine kinase and NRF2 enhances sensitivity to Paclitaxel in triple negative breast cancer

**DOI:** 10.1186/s13046-025-03625-y

**Published:** 2026-01-20

**Authors:** Irene Taddei, Claudia Cirotti, Fabienne Lamballe, Olivier Castellanet, Flavio Maina, Vanessa Medici, Fabrizio Fierro, Giacomo Corleone, Francesca De Nicola, Maurizio Fanciulli, Eleonora Cesari, Alba Di Leone, Alessia Piermattei, Angela Santoro, Chiara Naro, Claudio Sette, Daniela Barilà

**Affiliations:** 1 PhD Program in Cellular and Molecular Biology, Department of Biology, University of Rome “Tor Vergata”, Rome, Italy; 2 Laboratory of Cell Signalling, IRCCS-Fondazione Santa Lucia, Rome, 00179 Italy; 3 Department of Biology, University of Rome “Tor Vergata”, Rome, 00133 Italy; 4 Centre de Recherche en Cancérologie de Marseille (CRCM), Turing Center for Living Systems, Aix Marseille Univ, CNRS, INSERM, Institut Paoli-Calmettes, Marseille, 13009 France; 5 GSTeP Organoids Research Core Facility, Fondazione Policlinico Universitario Agostino Gemelli IRCCS, Largo Agostino Gemelli, Rome, 00168 Italy; 6 Gene Expression and Cancer Models Unit, IRCCS Regina Elena National Cancer Institute, Rome, Italy; 7 Breast Unit, Department of Women, Children and Public Health Sciences, Fondazione Policlinico Universitario Agostino Gemelli IRCCS, Roma, 00168 Italy; 8 Pathology Unit, Department of Woman and Child’s Health and Public Health Sciences, Fondazione Policlinico Universitario Agostino Gemelli, IRCCS, Rome, 00168 Italy; 9 Pathology Institute, Catholic University of Sacred Heart, Rome, 00168 Italy; 10 Department of Neuroscience, Section of Human Anatomy, Catholic University of the Sacred Heart, Largo Francesco Vito 1, Rome, 00168 Italy

**Keywords:** Triple Negative Breast Cancer, NRF2, MET, Receptor Tyrosine Kinase, Tyrosine Kinase Inhibitors, Therapy resistance, Paclitaxel, Patient-derived organoids

## Abstract

**Background:**

Triple-negative breast cancer (TNBC) is a very aggressive and heterogeneous cancer. The lack of effective targeted therapies and the frequency of relapses point to the urgent need to identify molecular vulnerabilities to overcome resistance to chemotherapy. Nuclear Factor Erythroid 2-related factor 2 (NRF2) is a transcription factor that plays a central role in response to oxidative stress. Its hyperactivation contributes to metabolic rewiring and resistance to therapy in several tumours including TNBC. Efficient pharmacological approaches that block NRF2 functions are still missing. Protein Tyrosine Kinases (PTKs), often overactivated in cancer and influencing several signalling pathways, are promising candidates to explore for their potential impact on NRF2.

**Methods:**

The link between Receptor Tyrosine Kinases (RTKs) and NRF2 expression and its impact on the survival probability of TNBC and non-TNBC patients were investigated by bioinformatic analyses using TCGA and GEO databases. MET-NRF2 connection was further confirmed by immunoblotting, immunofluorescence, RT-qPCR, and RNAseq experiments through the combinatorial use of murine and human TNBC cellular models. The efficacy of combination treatments with Paclitaxel and specific inhibitors of MET-NRF2 signalling was assessed by viability assays and flow-cytometry analyses on TNBC cellular models as well as on TNBC patient-derived organoids.

**Results:**

Here, we identify a novel interplay between MET and SRC kinases with NRF2 expression and activity and demonstrate that its targeting enhances the sensitivity to the standard Paclitaxel treatment of TNBC cells and patient-derived organoids.

**Conclusions:**

Our study shows that RTKs regulate NRF2 expression and activation in TNBC providing a proof of principle for the ability of Tyrosine Kinase Inhibitors (TKIs) to impinge on NRF2 signalling. Our findings also uncover the value of the MET-SRC-NRF2 axis as exploitable vulnerability in NRF2-hyperactivated TNBC, paving the way for the repositioning of TKIs as modulators of NRF2 signalling.

**Supplementary Information:**

The online version contains supplementary material available at 10.1186/s13046-025-03625-y.

## Background

Triple Negative Breast Cancer (TNBC) is one of the most aggressive invasive breast tumours. TNBC accounts for ∼15–20% of all the breast cancer (BC) cases and is associated to high heterogeneity and elevated metastatic potential [1]. At the molecular level, it is characterized by the absence of Estrogen Receptor (ER) and Progesterone Receptor (PR) expression, as well as the lack of amplification or overexpression of the Human Epidermal Growth Factor Receptor 2 (HER2) [2]. These characteristics prevent TNBC patients from responding to hormone therapy or anti-HER2 agents, limiting treatment options and resulting in a poor prognosis [3]. Indeed, the first line treatment remains chemotherapy followed by mastectomy and breast-conserving surgery, depending on the case and on the size of the tumour. Nevertheless, despite the initial chemosensitivity, TNBC patients show frequently relapses and metastasis, with more aggressive phenotypes than the primary tumour [4]. More recently, approaches based on immunotherapy [5] as well as on antibody-drug conjugates [6] have been introduced in the treatment of TNBC patients. Although these strategies have improved the therapeutic response, several challenges remain open encompassing the identification of novel biomarkers and the exploration of combined treatments [5, 6].

NRF2 (Nuclear factor-erythroid 2-related factor 2) is a Cap’n’collar nuclear transcription factor and it is considered the master regulator of oxidative stress response [7]. As an inducible transcription factor, its basal protein levels are tightly controlled and maintained at low concentrations through the association with its main negative regulator KEAP1 protein (Kelch-like ECH-associated protein 1), which forms the KEAP1-CUL3-RBX1 E3-ubiquitin ligase complex, thus mediating NRF2 ubiquitination and degradation via 26 S proteasome [8]. Oxidative stress and/or electrophiles can modify reactive Cys residues on KEAP1 protein preventing NRF2/KEAP1 interaction, thus promoting NRF2 stabilization, nuclear translocation and activation [9]. In addition, autophagy and serine/threonine signalling can promote p62 phosphorylation on Ser349, mediating KEAP1 degradation and therefore allowing NRF2 upregulation [10]. NRF2 has a cytoprotective role from xenobiotics and oxidative stress and it regulates more than 200 genes involved in detoxification processes, cellular redox homeostasis, autophagy, apoptosis, cell survival and proliferation [9]. Although traditionally considered a tumour suppressor, its dual role in cancer has become increasingly evident over the years [11]. In this regard, during cancer development, NRF2 hyperactivation creates a suitable environment that protects cancer cells from reactive oxygen species (ROS) damage further supporting tumour growth and drug resistance [12]. NRF2 hyperactivation in cancer is well-established in several tumours including BC. Importantly, in BC, elevated NRF2 expression correlates with poorer overall survival (OS) and disease-free survival (DFS), suggesting its potential as a prognostic factor for BC patients [13]. Of note, although the *NFE2L2* (NRF2) and *KEAP1* genes are rarely mutated in BC, NRF2 is highly expressed suggesting that it can be modulated by other mechanisms [14].

Protein Tyrosine Kinases (PTKs), including Receptor Tyrosine Kinases (RTKs) and non-Receptor Tyrosine Kinases (nRTKs), are involved in several biological processes such as cell survival, proliferation, migration, and differentiation [15]. Although TNBCs are very heterogeneous, most of them are characterized by aberrant activation of different RTKs, such as MET (Hepatocyte Growth Factor [HGF] Receptor) and EGFR (Epidermal Growth Factor Receptor) [16]. Indeed, MET overexpression and activation have been also reported in BC development [17] and associated with basal-like phenotype and identified in 52% of TNBC [18, 19].

SRC, a nRTK, was the first proto-oncogene to be discovered and its aberrant activation has been found in several solid tumours, including BC [20]. Notably, the *SRC* gene is rarely mutated or amplified in cancer and its hyperactivation is mainly due to the constitutive activation of RTKs, which occurs in a large majority of tumours [21]. In this regard, SRC is considered a common node of various RTKs, including MET, culminating in the deregulation of different downstream signalling pathways [22].

Here, we provide new evidence that MET and SRC promote NRF2 expression and activation in human and murine TNBC cellular models. We also demonstrate that pharmacological inhibition of MET, SRC, or NRF2 enhances the sensitivity of TNBC cellular models and TNBC patient-derived organoids (PDOs) to Paclitaxel treatment, pointing to this newly identified signalling cascade as a valuable target for the development of more effective combinatorial therapies for TNBC patients.

## Methods

### Cell culture

#### Mouse cell lines

MGT cell lines, derived from *MMTV-R26*^*Met*^ mice [23], were grown in DMEM/F12 (Dulbecco’s modified Eagle’s media/F12, 1/1, Sigma-Aldrich) supplemented with: 10% foetal bovine serum (FBS, Sigma-Aldrich), 100 U/mL penicillin, 100 mg/mL streptomycin (P/S, Sigma-Aldrich), L-glutamine (2mM, Sigma-Aldrich), glucose (0,25%, Sigma-Aldrich), insulin (10 µg/mL, Sigma-Aldrich), transferrin (10 µg/mL, Sigma-Aldrich), sodium selenite (5 ng/mL, Sigma-Aldrich), hydrocortisone (0,5 µg/mL, Sigma-Aldrich), EGF (20 ng/mL, Sigma-Aldrich), and HGF (10 ng/mL, Thermo Fisher Scientific), at 37° in a 5% of CO₂ atmosphere. All MGT cells are routinely tested and confirmed negative for *Mycoplasma* contamination.

#### Human cell lines

Human non-TNBC (T47D, MDA-MB-361, and SKBR3) and TNBC cell lines (MDA-MB-231) were cultured in RPMI-1640 (Sigma-Aldrich) supplemented with 10% FBS, L-glutamine (2mM,), and P/S. BT-549 (TNBC cell line) were cultured in DMEM with 10% FBS, L-glutamine (2mM), and P/S. MCF10-A cells, a non-transformed human mammary epithelial cell line, were grown in DMEM/F12 supplemented with horse serum (5%, Sigma-Aldrich), EGF (20 ng/mL), hydrocortisone (0,5 µg/mL), cholera toxin (100 ng/mL, Sigma-Aldrich), insulin (10ng/mL), and P/S. All cell lines are cultured at 37° in a 5% of CO₂ atmosphere and negatively tested for *Mycoplasma* contamination.

### Transient transfection experiments

For transient silencing of MET expression (MGT-13 and BT-549) and NRF2 expression (MGT-13), cells were seeded the day before and transfected for 24 h with Lipofectamine™ RNAiMAX Transfection Reagent (Invitrogen) by using murine and human MET siRNA (Santa Cruz Biotechnology; sc-35924, sc-29397) or NRF2 siRNA (Santa Cruz Biotechnology; sc-37049) according to the manufacturer’s instructions. For transient transfection, MGT-13 cells were seeded the day before and transfected for 24 h with Lipofectamine™ 2000 Transfection Reagent (Invitrogen) by using empty pSGT, SRC^Y527F^ and SRC^K295M^ vectors according to the manufacturer’s instructions as previously described [24].

### Breast Cancer Patient-Derived Organoids (BC-PDO) culture

BC-PDOs were obtained as previously described [25]. Briefly, breast cancer tissue was finely chopped, washed with 10 mL AdDF+++ (Advanced DMEM/F12 containing 1× Glutamax, 10 mM HEPES, and antibiotics), and digested in 10 mL of AdDF+++ containing 4 mg/mL collagenase II and 5 µM RHO/ROCK pathway inhibitor (Y-27632, Tocris) on an orbital shaker at 37 °C for 1–2 h. The digested tissue suspension was mechanically disrupted by pipetting up and down 10 times, passed through a 100 μm filter pre-coated with AdDF+++ containing 0.1% BSA, and centrifuged at 490 × g for 5 min. The pellet was resuspended in 10 mL AdDF+++ and centrifuged again.The pellet was incubated with 2 mL red blood cell lysis buffer for 5 min at room temperature to eliminate erythrocytes, followed by washing with culture medium and pelleting at 490 × g. The resulting pellet was resuspended in 10 mg/mL of cold Cultrex growth factor-reduced BME type 2, and 40 µL drops of the BME-cell suspension were allowed to solidify on pre-warmed 24-well suspension culture plates at 37 °C for 30 min. After polymerization, 400 µL of BC organoid medium (AdDF+++, 0.5 mM A8301, 1× B27, 5 ng/mL EGF, 100 nM β-estradiol, 5 ng/mL FGF7, 20 ng/mL FGF10, 10 µM forskolin, 5 nM heregulin β1, 0.5 µg/mL hydrocortisone, 1.25 mM N-acetylcysteine, 10 mM nicotinamide, 100 ng/mL noggin, 100 µg/mL primocin, 10% R-spondin-conditioned medium, 1 mM SB202190, 5 µM Y-27632) was added to each well, and the plates were placed in a humidified incubator at 37 °C with 5% CO2. The medium was refreshed every 3 days.

Organoids were passaged every 1–2 weeks by incubation with Cultrex Organoid Harvesting Solution for 45 min at 4 °C to digest the BME, and then dissociated by enzymatic digestion with TrypLE Express (Gibco) for 7–10 min at 37 °C, followed by pipetting up and down several times. TrypLE Express activity was blocked by adding 10 mL of AdDF+++ and centrifuging at 490 × g. Organoid fragments were resuspended in cold BME and re-seeded as described above at a suitable ratio (1:1 to 1:6), allowing the formation of new organoids.

### Antibodies and drugs

Primary antibodies are used as follows: anti-NRF2 (D1Z9C) (12721; Cell Signalling Technology), anti-NRF2 (EP1808Y) (ab-62352; Abcam), anti-phospho-SRC (Tyr416) (2101; Cell Signalling Technology), anti-SRC (2108; Cell Signalling Technology), anti-phospho-MET (Tyr1234/35) (3126; Cell Signalling Technology), anti-MET (3127; Cell Signalling Technology), anti-heme oxygenase 1 (A-3) (sc-136960; Santa Cruz Biotechnology), anti-phospho-histone H2AX (Ser139) (9718; Cell Signalling Technology), anti-KEAP1 (G-2) (sc-365626; Santa Cruz Biotechnology), anti-KEAP1 (F-10) (sc-514914; Santa Cruz Biotechnology), anti-p62 (SQSTM1) (PM045; MBL International), anti-phospho-p62 (SQSTM1) (Ser349) (PM074; MBL International), anti-phospho-EGFR (Tyr1068) (3777; Cell Signalling Technology), anti-EGFR (LA22) (05–104, Sigma-Aldrich), anti-8-OHdG (sc-66036, Santa Cruz Biotechnology), anti-vinculin (13901; Cell Signalling Technology), anti-lamin A/C (sc-376248; Santa Cruz Biotechnology), anti-GAPDH (sc-47724; Santa Cruz Biotechnology), anti-β-Actin (3700, Cell Signalling Technology);

PHA-665,752 (S1070; TargetMol), Dasatinib (CDS023389; Sigma-Aldrich), ML-385 (S8790; Selleckchem), Paclitaxel (T7191; Sigma-Aldrich);

### Protein extract, nuclei/cytoplasm fractionation and western blot analyses

Total protein lysates were prepared using Buffer A (10 mM Hepes [pH 7.9], 10 mM KCl, 1.5 mM MgCl2, 0.5 mM DTT, 0.1% NP-40) or RIPA Buffer (50mM Tris-HCL pH 8.0, 150mM NaCl, 1% NP40, 12mM sodium deoxycholate) supplemented with 10 mg/ml Protease Inhibitor Cocktail-1 (P2714; Sigma-Aldrich), 10 mg/mL TPCK, 1mM phenylmethylsulfonyl fluoride, 25mM NaF, 1mM sodium orthovanadate, 25 mM β-glycerophosphate. Lysates were incubated for 20 min on ice, then sonicated, and centrifuged at 12,000 g at 4 °C for 20 min. For nuclei and cytoplasm fractionation, cells were lysed in Buffer A (without NP-40) for 20 min on ice. NP-40 was then added to a final concentration of 0.1%. Next, nuclei were separated from the cytoplasm by centrifugation at 12,000 g at 4 °C for 30 s. The cytoplasm was harvested and the nuclear pellet was lysed in Buffer A supplemented with 0.05% NP-40 for 20 min on ice, then sonicated and centrifuged at 12,000 g for 20 min. For western blot, 30–80 µg of proteins were separated by SDS-PAGE, blotted on nitrocellulose membrane and incubated with specific antibodies.

### Immunofluorescence

Cells were seeded on coverslips and grown at 37 °C in a 5% CO₂ atmosphere. After treatments, cells were washed with 1X PBS and then fixed with 4% PFA for 15 min at room temperature (RT), permeabilized using PBS/Triton X-100 0.3% solution for 10 min. For γH2AX staining cells were additionally permeabilzed in cold 100% methanol for 10 min at −20 °C. Then, unspecific signals were blocked with BSA 3% in PBS solution for 1 h, and then incubated with primary antibodies (NRF2 1:50; γH2AX 1:300; p62 1:500, KEAP1 1:50) overnight in a humid chamber at 4 °C. Secondary antibodies (1:500, Thermo Fisher Scientific) were applied for 1 h at RT, and nuclei staining were performed using Hoechst 33342 (Thermo Fisher Scientific). The images were acquired with ZEISS fluorescence microscopy and analysed with ImageJ Fiji version 2.3. The images were acquired with confocal microscopy (Evident FV4000) with 60x oil objective. The open-source plugin ComDet 0.5.2 on ImageJ Fiji was used for the quantification of foci counts and colocalizing particles as previously described [24].

### 8-ox-DG immunofluorescence staining

Cells were seeded on coverslips and grown at 37 °C in a 5% CO₂ atmosphere. After treatments, cells were washed with 1X PBS and then fixed with 4% PFA for 15 min at room temperature (RT), permeabilized using PBS/Triton X-100 0.1% solution for 5 min. Cells were additionally permeabilized with cold 100% methanol for 30 min at −20 °C. Then, cells were incubate with 2 N of HCl for 45 min at RT to denaturate DNA. The reaction was neutralized with 50mM of Tris-HCl pH 8.8 for 5 min at RT. Unspecific signals were blocked in PBS 1X/BSA 3% for 30 min at RT, then, incubated with primary antibody anti-8-OHdG (1:50) overnight in a humid chamber at 4 °C. Secondary antibody (1:500, Thermo Fisher Scientific) was applied for 1 h at RT, and nuclei staining were performed using Hoechst 33342 (Thermo Fisher Scientific). The images were acquired with confocal microscopy (Evident FV4000) with 60x oil objective. The quantification of foci count/cell was analysed with the open-source plugin ComDet 0.5.2 on ImageJ Fiji. Particles size: 4 pixel.

### Histological analysis and immunohistochemistry (IHC)

The formalin fixed paraffin-embedded (FFPE) tissue blocks were collected and cut into 3–4 μm sections and mounted on Superfrost slides. The sections were analysed for MET (D-4) (sc-514148; Santa Cruz Biotechnology) and NRF2 (A-10) (sc-365949; Santa Cruz Biotechnology), both diluted at 1:100. Negative and positive control staining versus reactivity with the monoclonal antibodies was performed in each series. Positive/negative signal staining was assessed by an expert pathologist. The NRF2 (A-10) and MET (D-4) staining was evaluated as positive when more than 50% of tumor cells showed positive cell nuclei or cytoplasmic staining, respectively, relative to the negative controls.

### Real time PCR

Cells were homogenized with TRI Reagent (Themo Fisher Scientific) and RNA was extracted using the manufacturer’s protocol. One microgram of total RNA was retrotranscribed in cDNA using SensiFAST cDNA Synthesis KIT (Bioline). Specific pair of primers were designed and tested with primerBLAST. RT-PCR were performed using the SensiFAST Syber Low-ROX kit (Bioline) QuantStudio 3 RT–qPCR (Applied Biosystems). Data were analyzed using the second-derivative maximum method. The fold change in mRNA levels was compared to the control condition after normalization to the actin housekeeping gene.

List of human primers:

**Table:** 

GENE	FORWARD PRIMER	REVERSE PRIMER
*ACTIN*	5’-GGCCGAGGACTTTGATTGCA-3’	5’-GGGACTTCCTGTAACAACGCA-3’
*SQSTM-1*	5’-GGGAAAGGGCTTGCACCGGG-3’	5’CTGGCCACCCGAAGTGTCCG-3’
*HMOX1*	5’-CACAGCCCGACAGCATGCCC-3’	5’-GCCTTCTCTGGACACCTGACCCT-3’
*NQO1*	5’-GGTTTGGAGTCCCTGCCATT-3’	5’-CCTTCTTACTCCGGAAGGGTC-3’
*GCLC*	5’-CGCACAGCGAGGAGCTTCGG-3’	5’-CTCCACTGCATGGGACATGGTGC-3’
*SLC2A1*	໿5’-TCACTGTCGTGTCGCTGTTT-3’	໿5’-GGCCACGATGCTCAGATAGG-3’
*18 S*	5’-GGCCGTTCTTAGTTGGTGGA-3’	5’-TCAATCTCGGGTGGCTGAAC-3’
*NFE2L2*	5’-TTCCCGGTCACATCGAGAG-3’	5’-TCCTGTTGCATACCGTCTAAATC-3’

List of murine primers:

**Table:** 

GENE	FORWARD PRIMER	REVERSE PRIMER
*Actin*	5’-CACACCCGCCACCAGTTCGC-3’	5’-TTGCACATGCCGGAGCCGTT-3’
*Sqstm-1*	5’-GCTCTTCGGAAGTCAGCAAACC-3’	5’-GCAGTTTCCCGACTCCATCTGT-3’
*Hmox1*	5’-CACTCTGGAGATGACACCTGAG-3’	5’-GTGTTCCTCTGTCAGCATCACC-3’
*Nqo1*	5’-TGGCCGATTCAGAGTGGCATCCT-3’	5’-TGCATGCGGGCATCTGGTGG-3’
*Gclc*	5’-ACACCTGGATGATGCCAACGAG-3’	5’-CCTCCATTGGTCGGAACTCTAC-3’
*Slc2a1*	5’-GCTTCTCCAACTGGACCTCAAAC-3’	5’-ACGAGGAGCACCGTGAAGATGA-3’
*Nfe2l2*	5’-CTGAACTCCTGGACGGGACTA-3’	5’-CGGTGGGTCTCCGTAAATGG-3’

### Transcriptomic experiment and data analysis

Total RNA was extracted using Qiazol (Qiagen, IT), purified from DNA contamination through a DNase I (Qiagen, IT) digestion step and further enriched by Qiagen RNeasy columns for gene expression profiling (Qiagen, IT). Quantity and integrity of the extracted RNA were assessed by NanoDrop Spectrophotometer (NanoDrop Technologies, DE) and by Agilent TapeStation (Agilent Technologies, CA), respectively. RNA libraries for sequencing were generated using the same amount of RNA for each sample according to the Illumina Stranded Total RNA Prep kit with an initial ribosomal depletion step using Ribo-Zero Plus (Illumina, CA). The libraries were quantified by qPCR and sequenced in paired-end mode (2 × 100 bp) with NovaSeq 6000 (Illumina, CA). For each sample generated by the Illumina platform, a pre-process step for quality control was performed to assess sequence data quality and to discard low-quality reads.

RNA-seq data were analyzed with the nf-core tool version 3.3, using the “rnaseq” pipeline and default parameters [26], aligning reads to the reference genome for Mus musculus GRCm38. The output from nf-core was then used as input for the R package DESeq2 [27] to calculate differential expression. Genes with total raw counts across all samples below 50 were excluded to reduce background noise and improve the robustness of differential expression analysis. Normalized counts were transformed using the variance stabilizing transformation (VST function). Pathway ontology analysis was performed with ShinyGO version 0.8 [28], using the list of upregulated (log2FoldChange > 0.7 compared to control) or downregulated (log2FoldChange < −0.7) genes with pvalue < 0.05 as input. Transcription factors analysis, including calculation of activation z-score, was conducted with Qiagen IPA [29], selecting only those transcription factors with activation z-score greater than 2 or lower than − 2. The list of genes affected by NRF2 expression was also derived from IPA. Volcano plot and bar plots were generated with the ggplot package in R, PCA was performed using the plot PCA function from DESeq2, and heatmaps were created with the ComplexHeatmap package.

### Cell viability assay

MGT-13 and BT-549 cells were seeded in 96-well plates at 3,000 cells per well (150 µl media/well). After 24 h, cells were treated with single or combined drugs at the indicated concentrations. Cell viability was detected using Cell Counting Kit-8 (CCK-8, TargetMol) reagent after 72 h of treatment and then the absorbance was read using TECAN Infinitive-200 PRO spectrophotometer. The results represent the mean value of at least three independent experiments done in triplicates.

### Breast Cancer Patient-Derived Organoids (BC-PDO) viability assays

For PDO viability assays, organoids were harvested and dissociated as previously described and then resuspended in 2% BME growth medium before seeding in 100 µL volumes on BME-precoated 96-well plates. After 24 h, organoids were treated with the indicated drugs. After 5 days, the plate was imaged at 10× magnification using an IncuCyte SX5 live-content imaging system (Essen Bioscience) at 37 °C with 5% CO2. Viability was assessed using the CellTiter-Glo^®^ Luminescent Cell Viability Assay (Promega) with a microplate reader (Spark, Tecan). Drug dose–response curves were visualized using linear regression analysis in GraphPad Prism 9, and half-maximal inhibitory concentration (IC_50_) values were determined from the fitted curves. PDOs were classified as small, medium and large on their diameter measured manually drawing lines using ImageJ software. To calculate the organoid diameter range, we subtracted the diameter of the smallest organoid from the diameter of the largest organoid and divided this value by 3. Then, the obtained value was used to determine the diameter range for small, medium and large organoids [30].

### Clonogenic assay

Human TNBC cells were seeded in 35 mm dishes at 1,000 cells per well and incubated at 37 °C, 5% CO_2_ for colony formation. After 24 h, cells were treated with PHA-665752, ML-385 or Paclitaxel for 72 h. Each 2 days, the medium was changed until 10–15 days of growing. Then, colonies were fixed and stained with a solution containing 10% (vol/vol) methanol and 0.5% of crystal violet for 15 min for colony visualization. The stained colonies were counted. The results represent the mean value of at least three independent experiments.

### Cytofluorimetric analysis

Cell death analysis was evaluated upon combination treatments for 72 h by using a CytoFLEX S (Beckman Coulter) instrument. 1 × 10^6^ cells were collected and centrifuged at 300 g for 5 min at 4 °C and double-stained with Annexin V-APC-propidium iodide (PI) kit according to the manufacturer’s instructions (eBioscienceTM Annexin V Apoptosis Detection Kits; Thermo Fisher Scientific). PI was replaced with DAPI due to autofluorescence issues observed with some inhibitors. Unstained samples were used as control. Quality control was evaluated using CytoFLEX Daily QC Fluorospheres (Beckman Coulter). FCS files were analyzed using CytExpert version 2.2 software (Beckman Coulter). Cell death was represented as percentage to control condition.

### Reactive oxygen species measurement

Cells were incubated with 2.5 µM of Dihydroethidium (DHE) (Invitrogen) probe in complete medium for 20 min at 37 °C. Then, cells were collected, centrifuged at 500 g for 5 min at RT and resuspended in Hanks′ Balanced Salt solution (HBSS, Sigma-Aldrich). Fluorescence intensity was evaluated with CytoFLEX S (Beckman Coulter) instrument. Unstained samples were used as control. Quality control was evaluated using CytoFLEX Daily QC Fluorospheres (Beckman Coulter). FCS files were analyzed using CytExpert version 2.2 software (Beckman Coulter).

### Bioinformatics analysis of publicly available data of BC

For comparative gene expression analysis between BC subtypes, transcriptomic data (RNA sequencing) were obtained from The Cancer Genome Atlas (TCGA) database. Patients were stratified based on their estrogen receptor (ER), progesterone receptor (PR), and HER2 status. Gene correlation analysis was conducted using Spearman correlation coefficients, with statistical significance defined as *p* < 0.05. Volcano plot was done using the VolcaNoseR Rstudio package in R studio.

The GSE31519 cohort, comprising 579 TNBC patients with corresponding microarray data, was obtained from the Gene Expression Omnibus (GEO) database and used to generate Kaplan-Meier survival curves for TNBC. Patients were classified into high- and low- expression groups based on the median expression levels of the gene of interest. The statistical significance of survival differences was assessed using the log-rank test, and graphical representations were generated using GraphPad Prism software.

### Statistical analysis

All experiments represent the mean ± SEM or ± SD of at least three independent experiments (biological replicates). Drug interactions were evaluated using Combenefit software, which calculates the synergism score using Loewe additivity mathematical model based on viability parameters [23]. Negative values indicate antagonism, while positive values indicate additivity or synergism. The synergism score is calculated based on the combination index (CI) used to evaluate drug interactions. CI < 1 indicates synergism, CI = 1 means additivity and CI > 1 means antagonism. Error bars represent SD for immunofluorescence experiments and SEM for all the other techniques. Differences between data populations were assessed according to the two-tailed unpaired *t*-test (independent samples). For immunofluorescence analysis, an unpaired *t* test was used when data followed a normal distribution, and the Mann–Whitney test was applied in other situations. For multiple comparisons, we used the ANOVA test. Results were considered significant if *P*≤0.05 (*), *P*≤0.01 (**), *P*≤0.001 (***), *P*≤0.0001 (****). All statistical analyses were performed using GraphPad Prism software 8.4.2 version.

## Results

### Elevated MET/EGFR and NRF2 levels predict poor prognosis in TNBC

**Fig. 1:**
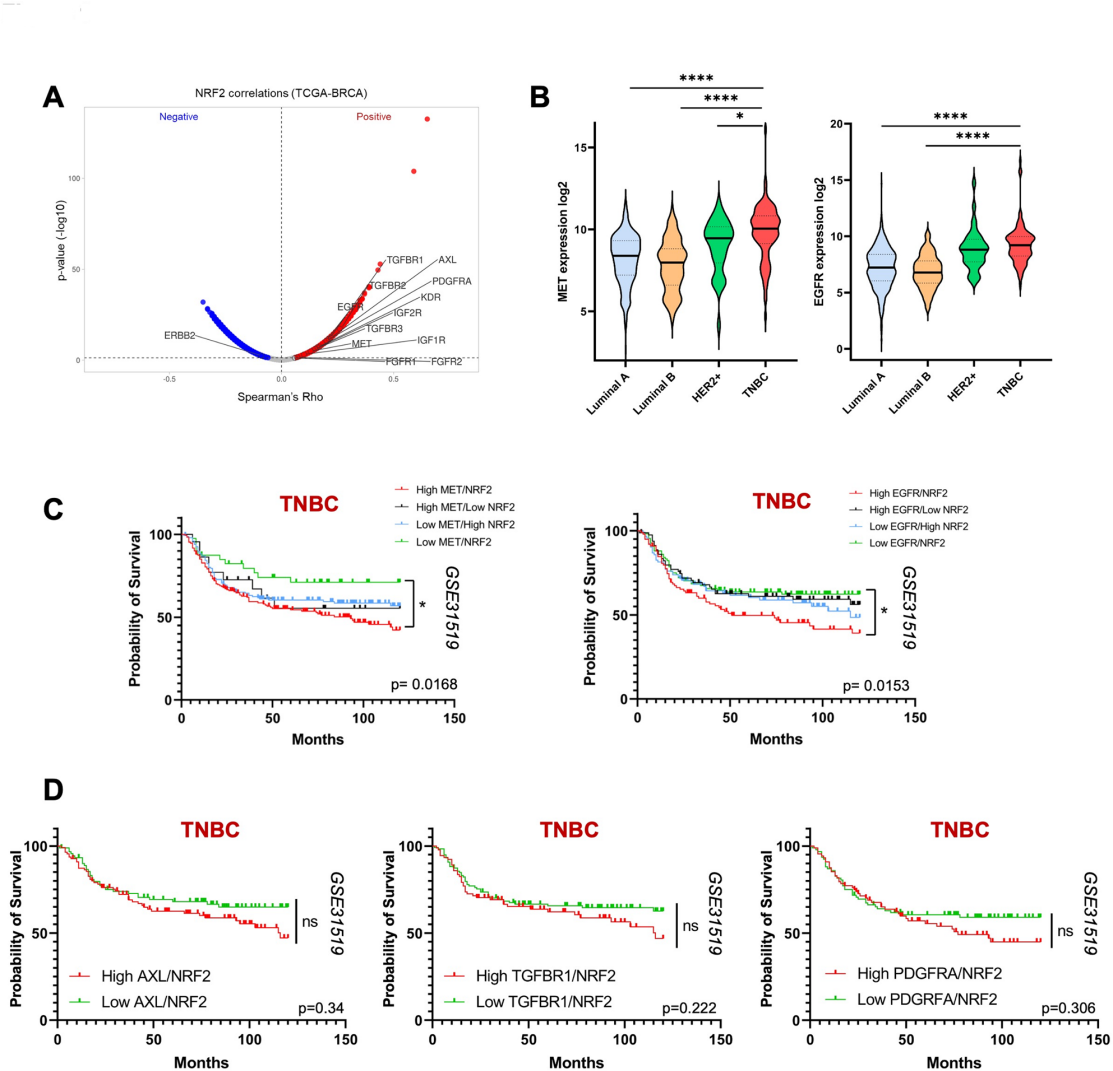
High expression levels of RTKs and NRF2 correlate with worst prognosis in TNBC patients. **A** Volcano plot for Spearman correlation between NRF2 expression and indicated RTKs in BC samples from TCGA-BRCA dataset. Significant positive correlations are shown in red, and negative correlations in blue. Common RTKs are highlighted. **B** Expression levels of *MET* and *EGFR* across the four different BC subtypes from the TCGA dataset. Kaplan–Meier curves showing the probability of overall survival in TNBC patients with different expression levels of *MET/NRF2* (left) and *EGFR/NRF2* (right) (**C**), *AXL*,* TGFBR1*,* PDGFRA*, and *NRF2* (**D**). Statistical analysis: (**B**) One-way ANOVA followed by Tukey’s multiple comparison statistical test was performed. **C-D** Survival data derived from GSE31519 dataset for TNBC patients. P values were computed using the Logrank (Mantel Cox) test. ns: not significant; * *p* < 0.05; ** *p* < 0.01; *** *p* < 0.001; **** *p* < 0.0001

RTKs are frequently overactivated in many tumours, including TNBC, and their constitutive activation results in aberrant signalling and deregulation of many transcription factors [31]. To test whether RTKs may play a role in NRF2 hyperactivation in TNBC, we first took advantage of the TCGA database, to perform a correlation analysis in BC patients, irrespective of the subtype. As show by the Spearman correlation analysis, the expression levels of *NFE2L2* (NRF2 gene name, here referred *NRF2*) positively correlate with the expression levels of several RTKs in BC (Fig. 1A). Of note, by evaluating the expression levels of different RTKs among the main four BC subtypes (Luminal A, Luminal B, HER2+, TNBC), we found that TNBC patients exhibit higher levels of *MET* and *EGFR* (Fig. 1B), a feature not observed for other RTKs such as *AXL*, *TGFBR1*, and *PDGFRA* (Fig.S1A). To further investigate this issue, and to get more insights on its relevance in TNBC, we evaluated whether the relative expression levels of different RTKs and *NRF2* influence the survival rates of TNBC patients. By using the GSE31519 dataset [32], we found a significant decrease in overall survival probability in patients who simultaneously express higher levels of *MET/NRF2* or *EGFR/NRF2* (Fig. 1C). Of note, a similar analysis performed in non-TNBC patients does not show any significant variation (Fig.S1B). By contrast, TNBC patients co-expressing high levels of *AXL/NRF2*,* TGFBR1/NRF2* or *PDGFRA/NRF2* were not characterized by alterations in overall survival (Fig. 1D). These data highlight a possible functional link between MET/EGFR and NRF2 in TNBC.

### MET targeting affects NRF2 signalling in TNBC murine model

**Fig. 2:**
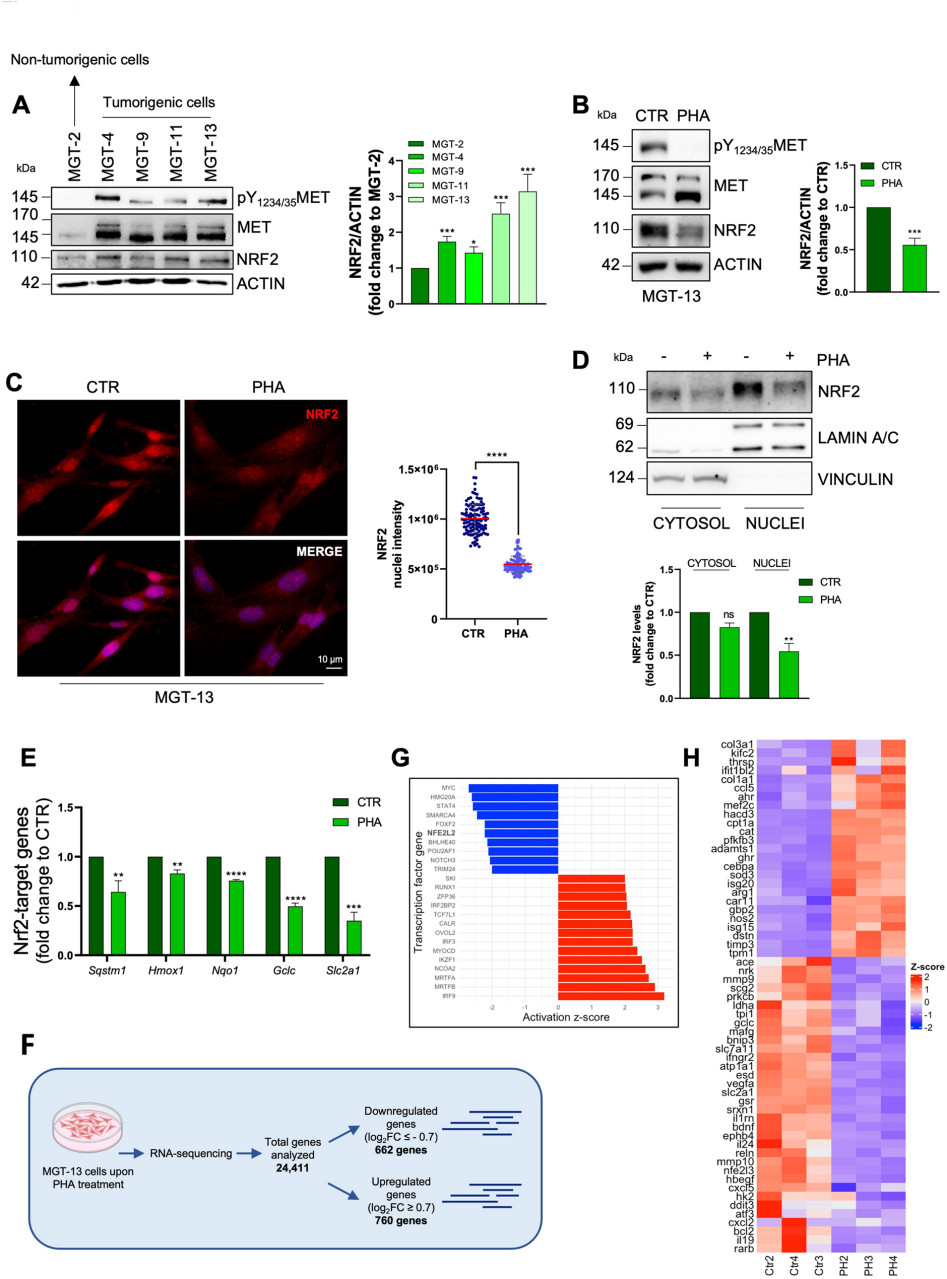
Inhibition of the MET receptor reduces NRF2 nuclear localization and activity in TNBC *MMTV-R26*^*Met*^ cell lines. **A** Immunoblotting (left) of pY_1234/35_MET, MET, NRF2, and relative densitometric analysis (right) of NRF2 protein levels in all MGT cell lines derived from the *MMTV-R26*^*Met*^ tumours. Actin was used as a loading control. **B** Immunoblotting (left) of pY_1234/35_MET, MET, NRF2, and relative densitometric (right) analysis of NRF2 protein levels in the MGT-13 cell line upon 16 h of PHA treatment. Actin was used as a loading control. **C** Immunofluorescence (left) and relative quantification (right) of NRF2 (red) nuclear intensity in MGT-13 cells upon 16 h of PHA treatment. DNA (Hoechst, blue). **D** Immunoblotting (top) and relative densitometric analysis (bottom) of NRF2 cytosolic and nuclear fractions in MGT-13 cells upon 16 h of PHA treatment. Vinculin and Lamin A/C were used as loading and quality controls. (**E**) RT-qPCR of NRF2 target genes in MGT-13 cells after 16 h of PHA treatment. Actin was used as housekeeping gene. **F** Workflow of RNA-seq experiments. p-value ≤ 0.5 and Log_2_foldchange (log₂FC) > 0.7 were used as threshold for analysis. **G** Activation z-score for transcription factors (CTRL vs. PHA). Values lower than − 2 (blue bars) suggest an inhibition of the pathway related to the corresponding transcription factor in the PHA treatment. Values over 2 (red bars) suggest an activation of the pathway in PHA samples. *NFE2L2* (NRF2) is indicated in bold. Data from IPA. **H** Heatmap of z-scores for genes involved in the NRF2 pathway (according to IPA) for CTRL vs. PHA (3 samples for each condition, see x axis). Positive values of z-score (red) indicate upregulation within the sample, negative values (blue) downregulation. Results represent the mean of at least three independent experiments (± SEM or ± SD). Statistical analysis: (**A-B-D**) Unpaired *t*-test. **C** Mann-Whitney test according to the normal distribution. **E** Multiple *t-* test. PHA: PHA-665752 1 µM, MET inhibitor. ns: not significant; * *p* < 0.05; ** *p* < 0.01; *** *p* < 0.001; **** *p* < 0.0001

To further investigate the significance of the interplay between RTKs and NRF2 in TNBC, we focused our studies on MET receptor. We took advantage of a unique mouse model, the *MMTV-R26*^*Met*^ mice, in which a slight increase in the expression levels of the *wild-type* form of MET in the mammary gland leads to the spontaneous development of BC [33]. This murine model represents an ideal system to investigate the role of MET in TNBC as all the developed tumours have been previously characterized as TNBC [33]. Furthermore, mammary gland tumour (MGT) cell lines that recapitulate the heterogeneity of the developed TNBC tumours have also been generated and characterized [33]. Immunoblotting analysis performed using a phosphospecific antibody (pY_1234/35_MET), which selectively recognizes the activated form of MET, showed the activation of MET in all four tumorigenic MGT cell lines (MGT-4, MGT-9, MGT-11, and MGT-13) compared to the non-tumorigenic MGT-2 cells (Fig. 2A). Notably, the tumorigenic MGT cells also showed higher levels of NRF2 expression, suggesting that MET hyperactivation sustains NRF2 signalling (Fig. 2 A). Consistent with this hypothesis, pharmacological inhibition of MET with PHA-665752, a specific inhibitor of MET activity, significantly decreased total NRF2 protein levels in MGT-13 cells (Fig. 2B). Interestingly, PHA-665752 treatment slightly reduced also EGFR activity as revealed by immunoblotting analysis using a phosphospecific antibody (pY_1068_EGFR), which recognized the activated form of EGFR (Fig.S2A), as previously reported [33]. Importantly, MGT-13 cells transiently silenced for MET expression (siMET), confirmed the downregulation of NRF2 levels as well as p62 and HO-1, two well-known NRF2 targets, similarly to what observed with PHA-665752 treatment (Fig.S2B-C). In addition, immunofluorescence analyses (Fig. 2 C, S2D) and subcellular fractionation (Fig. 2D) showed that both pharmacological (PHA-665752 treatment) and genetic silencing of MET expression (siMET) caused a significant decrease of NRF2 nuclear fraction. Lastly, pharmacological and genetic inhibition of MET expression hampered NRF2 transcriptional activity, as indicated by the reduced expression of well-known NRF2 target genes such as *sequestome-1 (SQSTM1)*,* heme oxygenase 1 (HMOX1)*,* NAD(P)H quinone dehydrogenase 1 (NQO1)*,* glutamate-cysteine ligase catalytic subunit (GCLC)* and *solute carrier family 2 (SLC2A1)* (Fig. 2E, S2E).

To further strengthen the link between MET and NRF2-dependent transcriptional activity, we performed a transcriptomic analysis on MGT-13 cells treated or not with PHA-665752. As expected, the treatment efficiently inhibits MET activity (Fig.S2F). Principal Component Analysis (PCA) shows that biological replicates of control and PHA-665752-treated samples are highly reproducible and segregate distinctly, according to their transcriptomic profile (Fig.S2G). A total of 24,411 genes were analysed and, among them, 662 genes exhibiting marked downregulation (log_2_FC ≤ − 0.7; Table S1) and 760 upregulation (log_2_FC ≥ 0.7; Table S2) upon PHA-665752 treatment (Fig. 2 F, S2H). Coherently with MET inhibition, we observed a strong deregulation of pathways related to migration and invasion of cancer cells (i.e., Mucine type O-glycan biosynthesis, ECM-receptor interaction, and focal adhesion), and pathways related to inflammation (i.e., IL-17 signalling pathway and cytokine-cytokine receptor interaction) (Fig.S2I). However, we noticed also an upregulation of pathways involved in resistance mechanisms or pathway linked to tumour suppressor functions (Fig.S2J). In searching to identify a link between MET and NRF2, we next performed enrichment analysis to define transcription factors (TFs) modulated by MET inhibition. Interestingly, NRF2 was identified as one of the most downregulated TF by the PHA-665752 treatment (Fig. 2G), further confirming its cooperative role with MET. Despite a moderate but significant downregulation of the TF itself (Log_2_FC = −0.56 compared to Ctrl) (Fig.S2K), the activation z-score of NRF2 (<−2) suggests that its target genes are significantly altered by the treatment. Analysing the transcriptional output of NRF2-associated genes revealed two clear clusters: 34 genes are downregulated, while 25 are upregulated (Fig. 2H) as a result of NRF2 inhibition. Then, we wondered if there was an overlapping set of downregulated genes in PHA-665752 treated samples compared with NRF2 silencing (siNRF2) in the same cellular model, confirmed by a significant downregulation of NRF2 (Fig.S2L). Pathways affected in NRF2 silenced cells are known to be regulated by NRF2 (i.e. ferroptosis, amino acid biosynthesis, oxidative stress), furtherly validating the reliability of the obtained results (Fig.S2M). Remarkably, genes whose downregulation is related to the NRF2 downregulation in the PHA-665752 RNAseq, showed a reduced expression also upon NRF2 interference (Fig.S2N; Table S3). Overall, these results demonstrate that MET sustains NRF2 signalling in TNBC murine models.

### Pharmacological inhibition of MET perturbs NRF2 signalling in human TNBC cell lines and reduces their clonogenicity potential

**Fig. 3:**
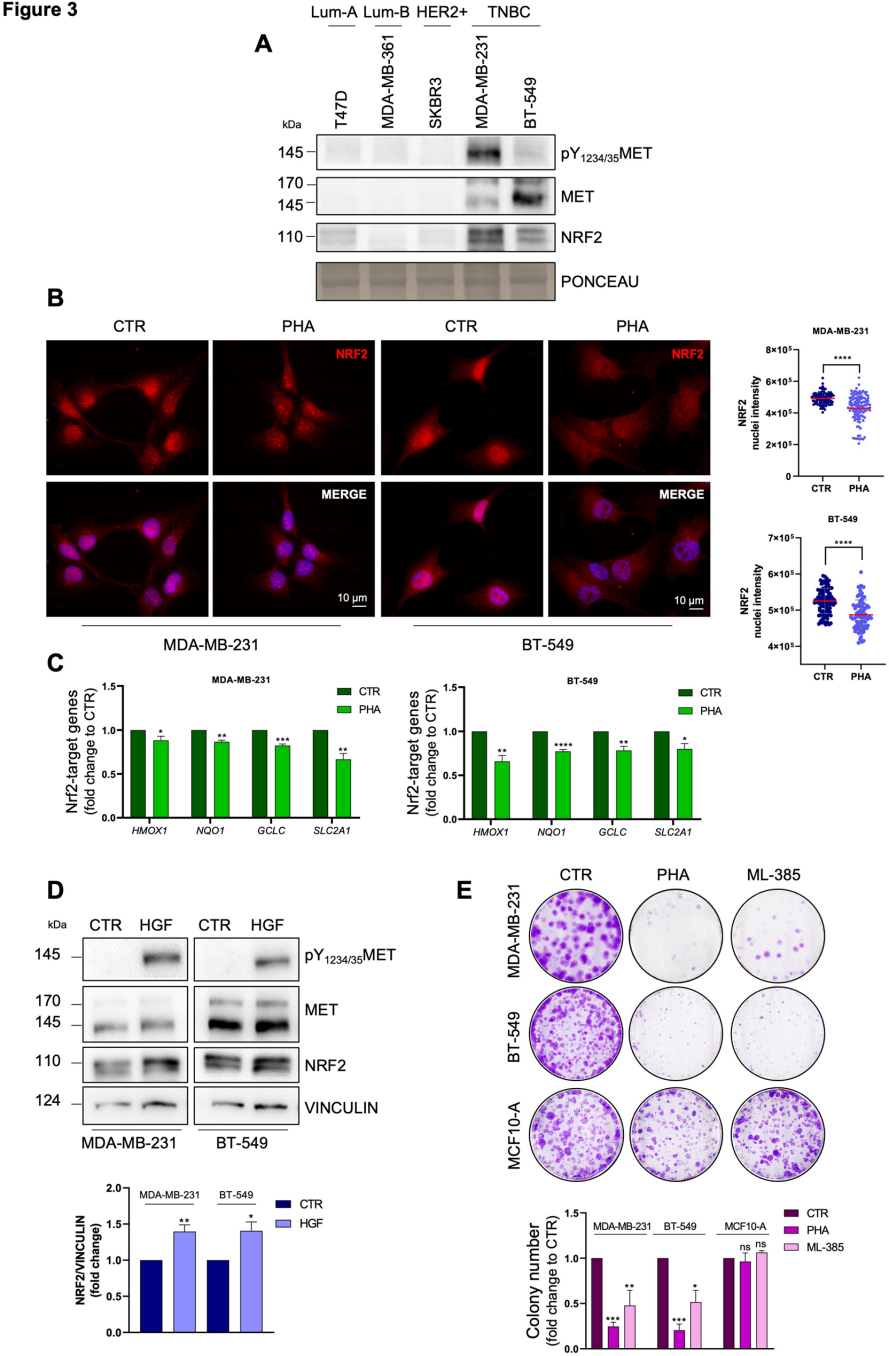
Targeting of the MET receptor reduces NRF2 activity in human TNBC cell lines. **A** Immunoblotting analysis of pY_1234/35_MET, MET, and NRF2 in human BC cell lines. Ponceau was used as a loading control. **B** Immunofluorescence (left) and relative quantification analysis (right) of NRF2 (red) nuclear intensity in human TNBC cells upon 16 h of PHA treatment. DNA (Hoechst, blue). **C** RT-qPCR of NRF2 target genes in human TNBC cells after 16 h of PHA treatment. Actin was used as housekeeping. **D** Immunoblotting (top) analysis of pY_1234/35_MET, MET, and NRF2 and relative densitometric analysis (bottom) of NRF2 in human BC cell lines after 4 h of serum-free media and 10 min of HGF stimulation (50ng/mL). Vinculin was used as a loading control. **E** Clonogenic assays (top) and relative quantification of the number of colonies (bottom) on MDA-MB-231, BT-549 and MCF10-A cells treated with PHA and ML-385 for 72 h. Results represent the mean of at least three independent experiments (± SEM or ± SD). Statistical analysis: (**B**) Mann-Whitney test according to the normal distribution. **C** Multiple *t*-test. **D** Unpaired *t*-test. **E** One-way ANOVA statistical test. PHA: PHA-665752 1 or 2 µM, MET inhibitor. ML-385: 5 µM, NRF2 inhibitor. ns: not significant; * *p* < 0.05; ** *p* < 0.01; *** *p* < 0.001; **** *p* < 0.0001

The above outcomes drove us to further explore the interplay between MET and NRF2 in human cellular models. First, we took advantage of the gastric tumour cell line GTL-16 characterized by *MET* amplification and overexpression and known to be addicted to MET signalling [34]. Immunoblotting analysis showed that, also in this system, MET inhibition by PHA-665752 strongly reduced NRF2 expression (Fig.S3A). This was accompanied by a downregulation of several NRF2 target genes, as shown by RT-qPCR (Fig.S3B), further strengthening the solidity of the newly identified axis MET-NRF2, even in a different cancer type. Next, we focused on human BC cellular models, utilizing cell lines belonging to different subtypes: T47D (Luminal-A), MDA-MB-361 (Luminal-B), SKBR3 (HER2+), MDA-MB-231 and BT-549 (TNBC). As shown by immunoblotting analysis, TNBC cell lines express higher levels of MET and NRF2. (Fig. 3 A). Importantly, immunofluorescence analyses on MDA-MB-231 and BT-549 cells revealed a decrease in NRF2 nuclear intensity following PHA-665752 treatment, confirming that MET inhibition (Fig.S3C) reduced NRF2 nuclear localization (Fig. 3B). Moreover, NRF2 transcriptional activity was affected upon MET inhibition in both cell lines (Fig. 3 C). Same results were obtained in BT-549 cells genetically silenced for MET expression (siMET) (Fig.S3D-G). Interestingly, we additionally found that the MET ligand Hepatocyte Growth Factor (HGF) significantly increased NRF2 expression and nuclear localization in both MDA-MB-231 and BT-549 cells, thereby confirming the link between MET and NRF2 (Fig. 3D, S3H).

To further evaluate whether pharmacological targeting of the MET-NRF2 axis may functionally affect human TNBC cells, we performed clonogenic assays. Pharmacological inhibition of MET with PHA-665752 and NRF2 with ML-385 (a specific inhibitor of NRF2 activity) significantly reduced the number of colonies formed by MDA-MB-231 and BT-549 cell lines, but not by MCF10-A, a non-tumorigenic human mammary cell line (Fig. 3E) [35]. Collectively, these data suggest that MET activity regulates NRF2 pathway also in human cellular models and that this axis may represent a specific therapeutic target in TNBC.

### MET and NRF2 targeting enhances sensitivity to Paclitaxel in TNBC cell lines

**Fig. 4:**
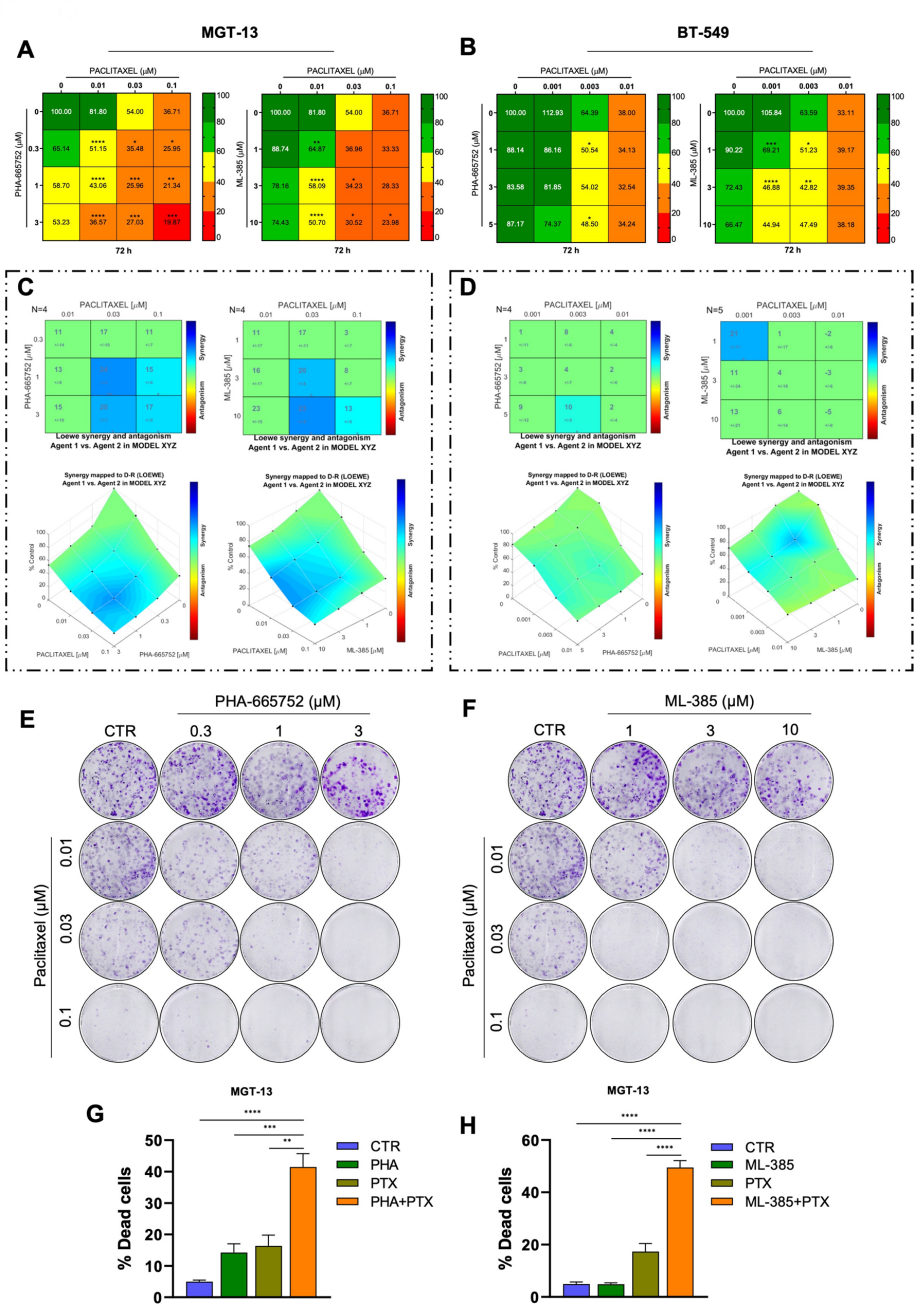
MET or NRF2 targeting enhances Paclitaxel sensitivity of TNBC cells. Cell viability of MGT-13 (**A**) and BT-549 (**B**) cells exposed to PTX in combination with PHA or ML-385. Percentage of cell viability in presence of drugs compared to controls (untreated cells) is indicated using a colour scale (from green to red). **C-D** Top panel: matrix synergy plot representing the synergy/antagonism score of each combination and its statistical significance calculated by Loewe model. Bottom panel: mapped to d-r surface showing the synergy distribution of drug combinations. The synergistic analysis was performed on MGT-13 (**C**) and BT-549 (**D**) cell lines. **E-F** Clonogenic assays on MGT-13 cells exposed to PTX in combination with PHA (**E**) or ML-385 (**F**) and treated as in Fig. 4A. **G-H** Histograms of flow cytometry experiments representing the percentage of dead cells upon ANNEXIN V/DAPI^+^ staining of MGT-13 cells exposed for 72 h to PHA and PTX (**G**) or ML-385 and PTX (**H**) treatments alone or in combination. Results represent the mean of at least three independent experiments (± SEM). Statistical analysis: (**A-B-G-H**) One-way ANOVA statistical test was performed for each combination compared to the drug alone. (In A-B, * indicate the significance respect to PTX treatment). **C-D** Loewe models were used for synergy score calculation by Combenefit software. PHA: PHA-665752: MET inhibitor; ML-385: NRF2 inhibitor. PTX: Paclitaxel. ns: not significant; * *p* < 0.05; ** *p* < 0.01; *** *p* < 0.001; **** *p* < 0.0001

The absence of ER, PR, and overexpressed HER2 prevents TNBC patients from responding to hormone therapy or HER2-targeted drugs, significantly limiting their treatment options to chemotherapy, with or without immunotherapy, radiotherapy, and surgery [3]. Furthermore, high heterogeneity and resistance to chemotherapy characterize many TNBC cases, resulting in poor prognosis [3]. For these reasons, uncovering novel molecular pathways that can be targeted is urgently needed. We therefore asked whether MET and NRF2 targeting may increase TNBC sensitivity to chemotherapy, beside reducing clonogenicity potential. To this aim, we performed cell viability assays both in murine and human TNBC models by treating either MGT-13 or BT-549 cells with PHA-665752 (MET inhibitor) or ML-385 (NRF2 inhibitor) in combination with Paclitaxel (PTX), a chemotherapeutic agent commonly used in the clinic for TNBC patients. As shown by the heatmap reporting the percentage of survival cells, the combination of PHA-665752 or ML-385 with PTX significantly reduced cell viability in both cell lines (Fig. 4A-B). Using Combenefit software [23] to calculate the Loewe additivity score, we found that these combination treatments exert a synergistic effect (Fig. 4C-D). Remarkably, the combined treatments with either PHA-665752 or ML-385 with PTX displayed a stronger effect on clonogenicity compared to single treatments in both murine and human TNBC cells (Fig. 4E-F, S4A-B). Flow cytometry analysis revealed a significant increase of the percentage of MGT-13 cell death upon combined treatments with either PHA-665752 or ML-385 with PTX (Fig. 4G-H). Overall, these data strengthen the relevance of targeting MET-NRF2 axis to enhance chemotherapy sensitivity in TNBC.

### MET-NRF2 axis inhibition impairs the response to ROS and increases DNA damage accumulation

**Fig. 5:**
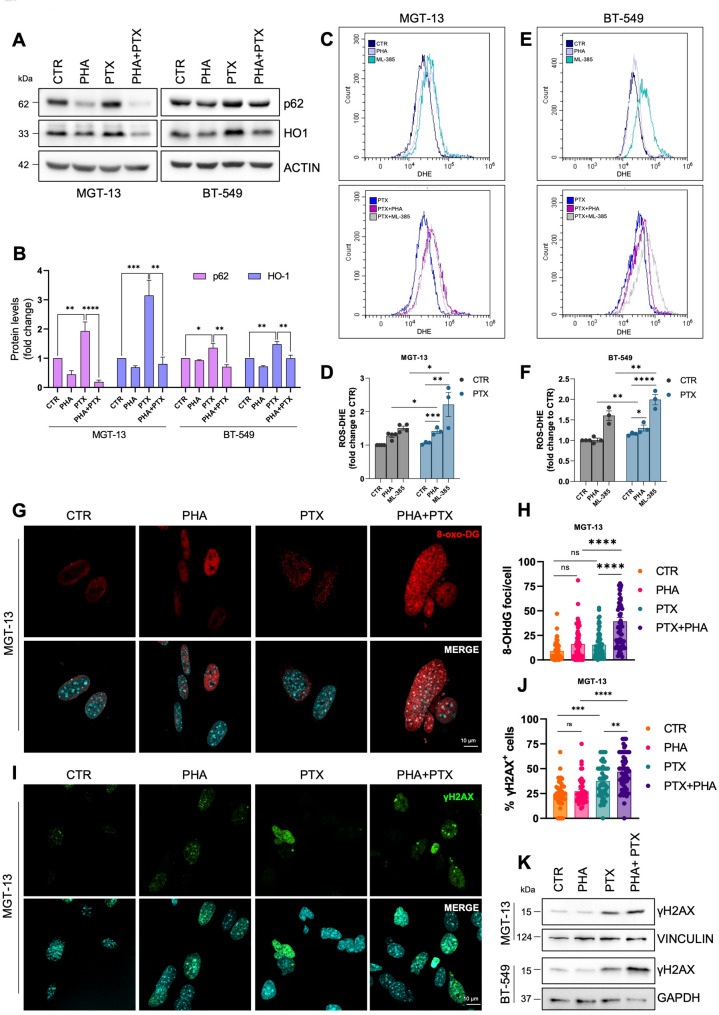
MET-NRF2 axis inhibition increases ROS levels and DNA damage accumulation. Immunoblotting (**A**) and relative densitometric analysis (**B**) of p62 and HO-1 in MGT-13 and BT-549 cells exposed for 24 h to PTX in combination with PHA. Actin was used as a loading control. **C-F** Cytofluorimetric analyses (top) and relative histograms (bottom) of reactive oxygen species (ROS) upon DHE staining on MGT-13 (**C-D**) and BT-549 (**E-F**) cells exposed for 24 h to PTX in combination with PHA or ML-385. Confocal microscopy analysis (**G**) and relative quantification (**H**) of 8-oxo-DG (red) foci/cell in MGT-13 cells treated alone or in combination with PTX and PHA. DNA (Hoechst, blue). Confocal microscopy analysis (**I**) and relative quantification (**J**) of γH2AX (green) positive cells in MGT-13 cells treated alone or in combination with PTX and PHA. **K** Immunoblotting analysis of γH2AX in MGT-13 and BT-549 treated as in (**I**). Vinculin and GAPDH were used as loading control. Results represent the mean of at least three independent experiments (± SEM or ± SD). Statistical analysis: (**B-D-F-H-J**) One-way ANOVA statistical test was performed for each combination compared to the drug alone. PHA: PHA-665752 1 or 3 µM, MET inhibitor. ML-385: 5 µM, NRF2 inhibitor. PTX: Paclitaxel 3nM or 30nM. ns: not significant; * *p* < 0.05; ** *p* < 0.01; *** *p* < 0.001; **** *p* < 0.0001

To clarify how the inhibition of MET-NRF2 axis may ameliorate the sensitivity to PTX, we first investigated the effect of PTX on NRF2 expression and activity in both MGT-13 and BT-549 cells. We found that PTX treatment increased the expression of NRF2, HO-1 and p62 proteins, as well as of a subset of NRF2 target genes (Fig.S5A-D). Immunofluorescence experiments highlighted an increase in NRF2 nuclear localization upon PTX treatment (Fig.S5E). These results are consistent with previous studies that pointed to NRF2 upregulation as a well-known mechanism to establish chemoresistance [36]. Indeed, it has been reported that in response to PTX, cancer cells enhanced NRF2 expression to evade PTX-induced cell death [36]. Remarkably, PHA-665752 impaired p62 and HO-1 upregulation in response to PTX (Fig. 5A-B), suggesting that MET inhibition may sensitize cells to PTX by dampening NRF2 activity.

NRF2 is the master regulator of oxidative stress response, and its activation is well-known to scavenge ROS accumulation in response to different stimuli [7]. Consistently, we could show that the pharmacological inhibition of NRF2 significantly enhanced ROS accumulation both in human and in murine cells co-treated with PTX plus ML-385 or PHA-665752 (Fig. 5C-F). In line with this, we also observed the accumulation of oxidative lesions to DNA, as showed by 8-oxo-2’-deoxyguanosine (8-oxo-DG) foci accumulation upon PHA-665752 and PTX co-treatment (Fig. 5G-H, S5F). We therefore sought to investigate whether MET inhibition may enhance the DNA damage accumulation induced by PTX. PTX alone induced DNA damage as revealed by the detection of γH2AX, a well-known marker of DNA damage, as shown by immunofluorescence and immunoblotting analyses in both murine and human cells, which was further increased by co-treatment with PHA-665752 (Fig. 5I-K, S5G), consistently with the increased toxicity shown in Fig. 4.

Overall, these data document a role for MET-NRF2 axis in promoting PTX resistance and suggest that its targeting could be a valuable strategy to increase oxidative stress and DNA damage, thereby enhancing cell sensitivity.

### MET modulates NRF2 signalling through SRC kinase-dependent p62/KEAP1 pathway

We then asked how MET activity may modulate NRF2 expression and signalling. We recently reported that the nRTK SRC triggers NRF2 hyperactivation and ferroptosis resistance in glioblastoma cellular models [24]. Indeed, SRC sustains mTORC1-dependent p62 phosphorylation on Ser349, driving its interaction with KEAP1 and allowing the release of NRF2-KEAP1 binding, thus resulting in NRF2 stabilization [24].

**Fig. 6:**
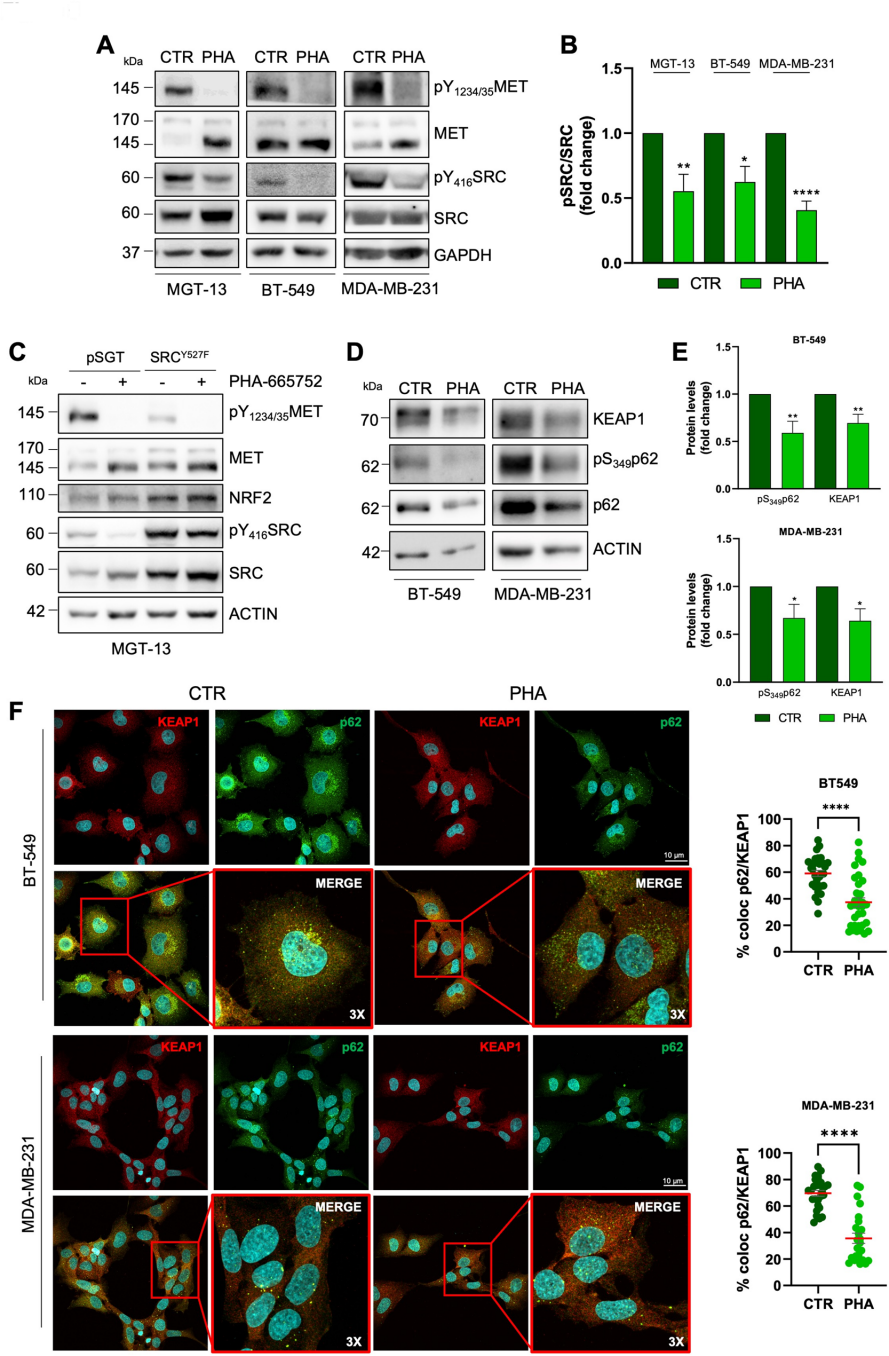
MET modulates NRF2 signalling via SRC kinase-dependent p62/KEAP1 pathway. Immunoblotting (**A**) of pY_1234/35_MET, MET, pY_416_SRC, SRC and relative densitometric analyses (**B**) of pY_416_SRC normalized on total SRC in TNBC cell lines after 16 h of PHA treatment. GAPDH was used as loading control. **C** Immunoblotting pY_1234/35_MET, MET, pY_416_SRC, SRC, and NRF2 in MGT-13 cells transiently transfected with empty vector (pSGT) and active SRC (SRC^Y527F^) after 16 h of PHA treatment. Actin was used as loading control. Immunoblotting (**D**) and relative densitometric analyses (**E**) of KEAP1, pS_349_p62 and p62 in human TNBC cell lines after 16 h of PHA treatment. Actin was used as loading control. **F** Confocal microscopy analyses and relative quantification of colocalizing dots of KEAP1 (red) and p62 (green) in human TNBC cell lines upon 16 h of PHA treatment. DNA (Hoechst, blue). 3X digital magnification showing merged signals. Results represent the mean of at least three independent experiments (± SEM or ± SD). Statistical analysis: (**B-E**) Unpaired *t*-test. **F** Mann-Whitney test according to the normal distribution. PHA: PHA-665752 1 µM, MET inhibitor. * *p* < 0.05; ** *p* < 0.01; *** *p* < 0.001; **** *p* < 0.0001

Of note, SRC hyperactivation is part of the signalling cascade activated downstream many RTKs, including MET [37]. We then tested the hypothesis that MET could drive NRF2 activation through SRC. We verified that the pharmacological and genetic inhibition of MET impinged on SRC activity in both human and murine TNBC cells, as revealed by pY_416_SRC antibody (Fig. 6A-B, S6A). The overexpression of the catalytically inactive mutant SRC^K295M^, reduced NRF2 expression levels (Fig.S6B-C) similarly to what observed with PHA-665752 treatment, supporting the link between MET-SRC and NRF2. Instead, the overexpression of the constitutively active mutant SRC^Y527F^, rescued the decrease of NRF2 expression levels triggered by PHA-665752 (Fig. 6 C).

In line with our previous findings, immunoblotting experiments revealed that p62 is highly phosphorylated on S349 in TNBC cells and the pharmacological inhibition of MET dramatically impinged on this phosphorylation (Fig. 6D-E, S6D-E). Moreover, confocal microscopy analyses revealed a strong colocalization between p62 and KEAP1 in both human and murine TNBC cells, which is severely reduced upon MET inhibition (Fig. 6 F, S6F), supporting the conclusion that MET impinges on NRF2 signalling through SRC-p62-KEAP1 axis.

### Pharmacological inhibition of SRC with dasatinib reduces NRF2 signalling

**Fig. 7:**
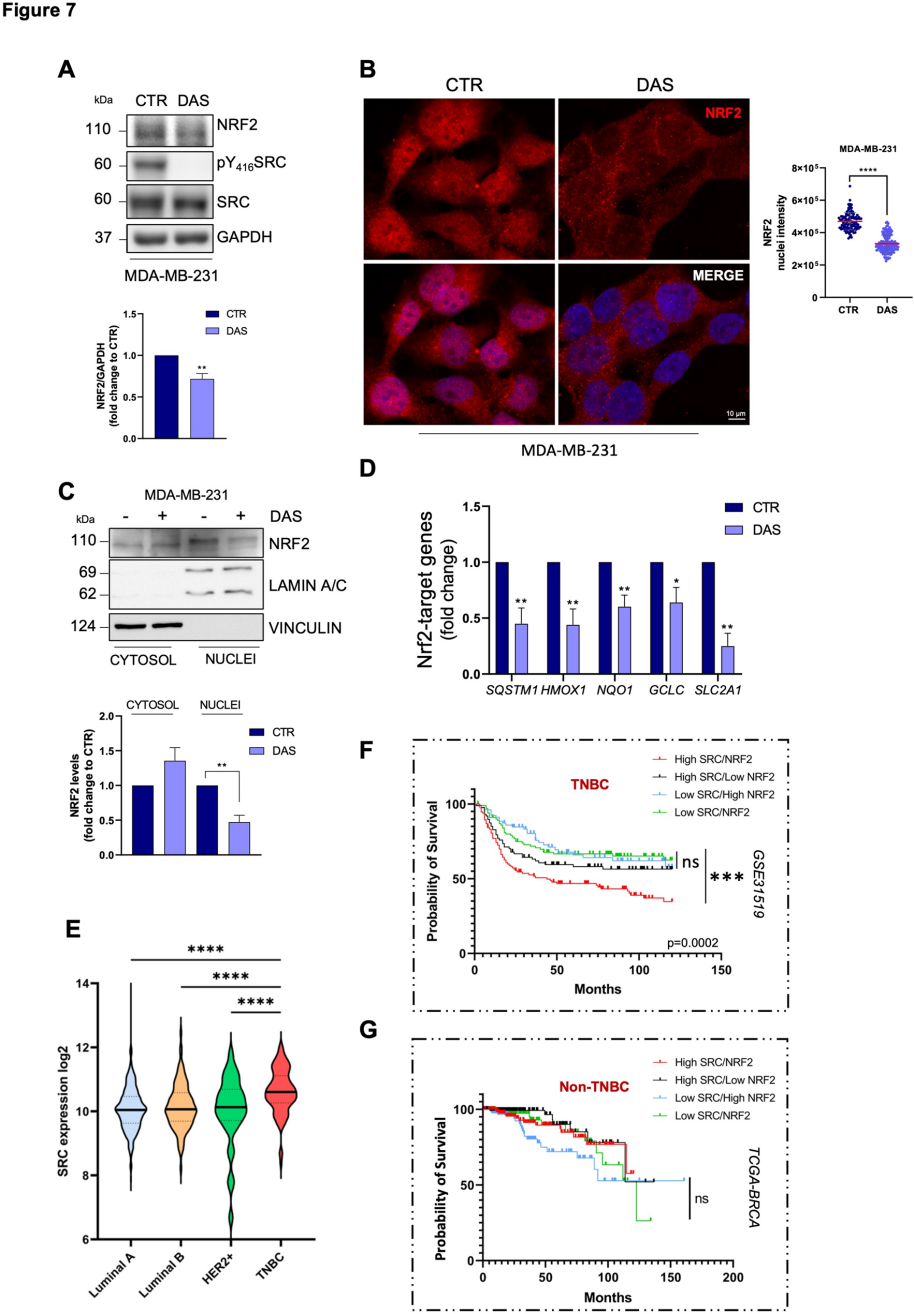
SRC expression sustains NRF2 nuclear accumulation and activity in TNBC. **A** Immunoblotting (top) of NRF2, pY_416_SRC, SRC and relative densitometric analysis (bottom) of NRF2 protein levels in MDA-MB-231 cells upon 16 h of DAS treatment. GAPDH was used as a loading control. **B** Immunofluorescence (left) and relative quantification (right) of NRF2 (red) nuclear intensity in MDA-MB-231 cells upon 16 h of DAS treatment. DNA (Hoechst, blue). **C** Immunoblotting (top) and relative densitometric analysis (bottom) of NRF2 cytosolic and nuclear fractions in MDA-MB-231 cells upon 16 h of DAS treatment. Vinculin and Lamin A/C were used as loading and quality controls. **D** RT-qPCR of NRF2 target genes in MDA-MB-231 cells after 16 h of DAS treatment. Actin was used as housekeeping gene. **E** Expression levels of *SRC* in log_2_ across the four different breast cancer subtypes from the TCGA dataset. **F-G** Kaplan–Meier curves show the overall survival probability of TNBC (*n* = 579; **F**) and non-TNBC (*n* = 544; **G**) patients with different expression levels of *SRC* and *NRF2*. Results represent the mean of at least three independent experiments (± SEM or ± SD). Statistical analysis: (**A-C**) Unpaired *t-*test. **B** Mann-Whitney test according to the normal distribution. **D** Multiple *t*-test. **E** One-way ANOVA followed by Tukey’s multiple comparison statistical test was performed. **F-G** Survival data obtained from Gene Expression Omninbus (GEO) Id GSE31519 for TNBC patients and TCGA dataset for non-TNBC patients. P values were computed using the Logrank (Mantel Cox). DAS: Dasatinib 50 nM; SRC inhibitor. ns: not significant; * *p* < 0.05; ** *p* < 0.01; *** *p* < 0.001; **** *p* < 0.0001

Having established that SRC activity may mediate NRF2 activation downstream of MET, we next asked whether pharmacological inhibition of SRC could affect NRF2 signalling in a similar manner to what observed with PHA-665752. Dasatinib (DAS), a well-known SRC kinase inhibitor, slightly but significantly reduced total NRF2 protein expression levels in human TNBC cell lines (MDA-MB-231 and BT-549; Fig. 7 A, S7A). Immunofluorescence analyses showed that treatment with DAS decreased NRF2 nuclear localization in both cell lines (Fig. 7B, S7B). Subcellular fractionation experiments also confirmed these results (Fig. 7 C). Coherently, SRC pharmacological inhibition strongly affected NRF2 transcriptional activity, as revealed by the downregulation of several NRF2 target genes detected by RT-qPCR analysis (Fig. 7D).

To further investigate the significance of SRC-NRF2 interplay in TNBC, we analysed *SRC* expression by querying TCGA datasets of BC samples. Among BC subtypes, TNBC exhibited the highest levels of *SRC* expression (Fig. 7E). Next, we evaluated the overall survival probability of TNBC patients stratified by different levels of *SRC* and *NRF2*, using the GSE31519 cohort [32]. Patients expressing simultaneously higher levels of *SRC* and *NRF2* are characterized by poorer clinical outcomes compared to patients with other expression profiles (Fig. 7 F). In contrast, the same analysis performed in non-TNBC patients revealed no significant differences (Fig. 7G).

All together, these data suggest that SRC kinase targeting may also represent a valuable strategy to dampen NRF2 signalling and therefore enhance Paclitaxel efficacy.

### Targeting MET-SRC-NRF2 axis improves chemotherapy efficacy in TNBC patient-derived organoids

**Fig. 8:**
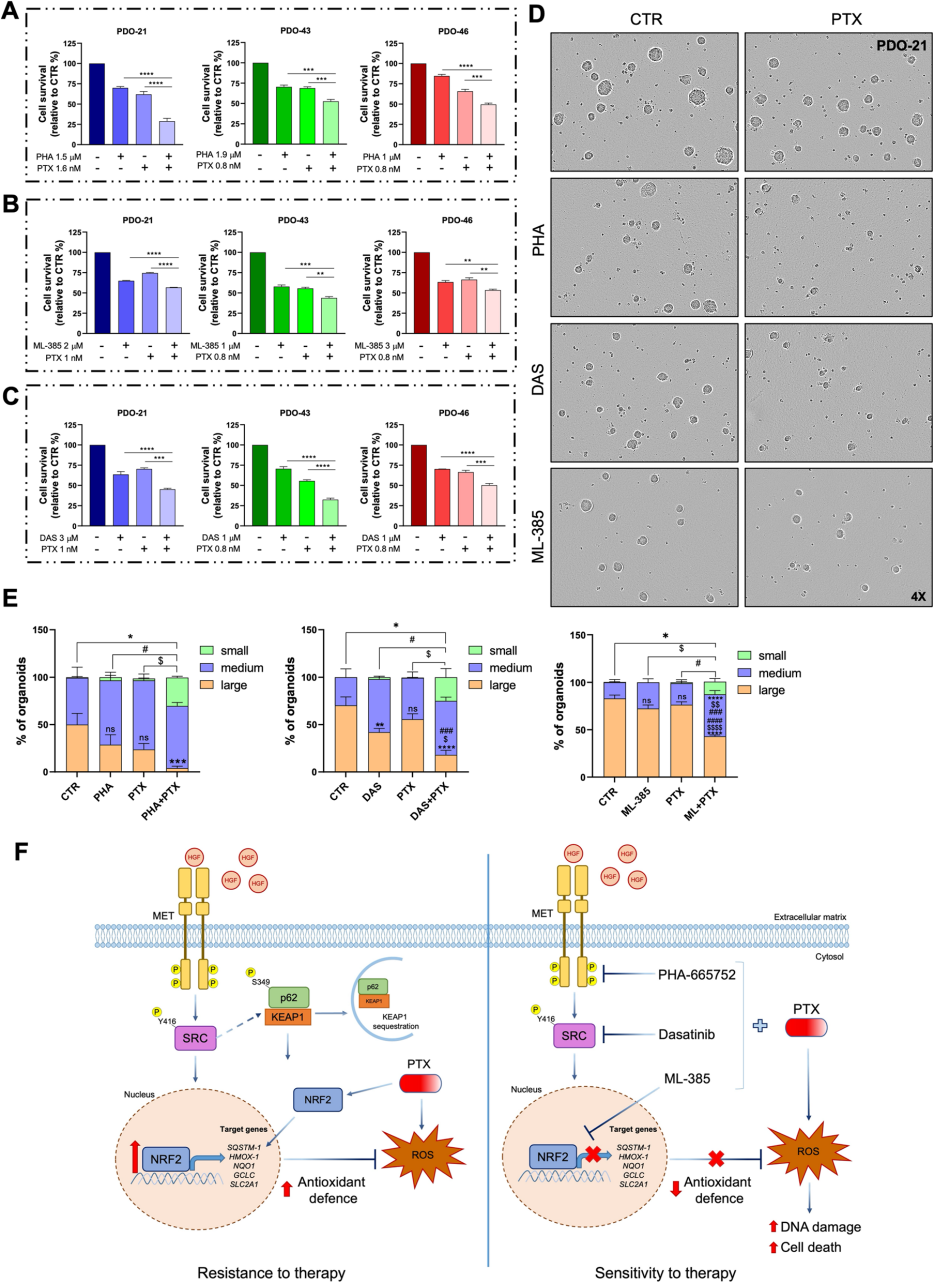
MET, SRC or NRF2 targeting improves Paclitaxel efficacy in TNBC patient-derived organoids. Organoid viability assays of PDO-21, PDO-43, and PDO-46 after PHA and PTX (**A**), ML-385 and PTX (**B**), DAS and PTX (**C**) combination treatments. **D** 4X digital magnification of bright-field images and relative quantification of organoids size of PDO-21 culture treated as described in (**A-B-C**). Scale bar: 400 μm. **E** Percentage of small, medium, and large PDO-21 treated as in (**A**-**B**-**C**). **F** Working model. Results represent the mean of at least three independent experiments (± SEM). Statistical analysis: (**A-B-C**) One-way ANOVA statistical test was performed for each combination compared to the drug alone. **E** Two-way ANOVA followed by Tukey’s multiple comparison test (* respect to CTR, $ respect to inhibitors and # respect to PTX). PHA: PHA-665752, MET inhibitor. DAS: Dasatinib. ML-385: NRF2 inhibitor. PTX: Paclitaxel. ns: not significant; *, $, # *p* < 0.05; **, $$, ## *p* < 0.01; ***, $$$, ### *p* < 0.001; ****, $$$$, #### *p* < 0.0001

To strengthen our discoveries in TNBC models that are closer to the clinical setting, we employed patient-derived organoids (PDOs). PDOs are human models that recapitulate the genetic and phenotypic feature of the tumour of origin, including the response to treatments [38]. We took advantage of three PDOs, named PDO-21, PDO-43, PDO-46 (Table S4), derived from tumours that do not exhibit amplification or mutations in the *SRC*, *NFE2L2* or *KEAP1* genes, previously characterized as faithful TNBC models [25]. We first determined the IC_50_ of PHA-665752, DAS, ML-385, and PTX in the three PDOs (Fig.S8A-B), then tested the effectiveness of combinatorial treatments with these drugs. We found that the combination of PTX with PHA-665752, ML-385, or DAS significantly reduced viability of PDOs (Fig. 8A-C), consistent with what was observed in TNBC cell lines. Additionally, the proposed combinations reduce the size of the PDOs that survived to the treatments, although with different efficacy (Fig. 8D-E, S8C-E). These results further support the link between MET and NRF2 in different TNBC preclinical in vitro models. Notably, the analysis on a small cohort of 15 TNBC biopsies by IHC, revealed that most of the analysed sections displayed MET expression as well as high levels of nuclear NRF2 positivity (Fig.S8F-G: panels C-D; Table S5). Of note, two samples negative for MET expression displayed a weaker NRF2 signal (Fig.S8F-G: panels A-B; Table S5). All together, these data are in agreement with our proposed model (Fig. 8 F) and suggest that targeting MET, SRC, or NRF2 can improve the chemotherapy efficacy of PTX and may therefore represent a promising therapeutic strategy for ameliorating TNBC therapy.

## Discussion

TNBC is a highly aggressive and heterogeneous disease, characterized by the absence of target therapy and poor prognosis [1, 3]. In this regard, the identification of new molecular pathways that drive TNBC resistance to therapy is urgently needed. NRF2 transcription factor, the master regulator of oxidative stress response [12], is aberrantly activated in several tumours including TNBC and it is associated with radio- and chemoresistance mechanisms [14]. NRF2 is considered an “undruggable” protein due to the lack of active sites or allosteric pockets [39]. Most of the NRF2 inhibitors used for research purposes are natural plant-derived compounds, like polyphenols. Although these are commonly referred to as antioxidants, some of them inhibit NRF2-dependent expression of cytoprotective genes [40]. The inhibitory effect of these natural compounds is still controversial and, although highly safe, they have a weak specificity [41, 42]. So far, ML-385 is the only selective inhibitor currently available that targets the ability of NRF2 to dimerize with MAFG, although not approved for clinical use [43]. More recently, it has been reported a novel NRF2 inhibitor, ARE-PROTAC chimeric molecule, which selectively degrades NRF2-MAFG heterodimer via ubiquitin-proteasome system [44]. Its potential use in the clinic has not been investigated yet. Thus, available compounds display several limitations, pointing to the urgent need to uncover molecular mechanisms regulating NRF2 expression and activity [39]. The identification of molecular mechanisms and signalling circuits responsible for NRF2 pathway activation in cancer that can be targeted with available agents represents an alternative strategy to dampen NRF2, implementing therapeutic options.

Genetic mutation on NRF2 (*NFE2L2*) or its major negative regulator *KEAP1* are responsible for NRF2 hyperactivation in several cancer types [11]. Still, NRF2 deregulation is independent of these mutations in others, illustrating the relevance of rewiring NRF2 signalling in tumorigenesis [11]. It has been recently reported that cysteine mediated NRF2 activation represents a novel survival mechanism for TNBC [45].

The deregulation of RTKs and nRTKs is a common feature in various types of cancer, including TNBC. Physiologically, their action is tightly controlled to ensure proper cellular proliferation, survival, migration, whereas their aberrant activation in cancer can be therapeutically exploited with the use of selective TKI blocking agents widely used in the clinic [46]. However, several clinical trials in different tumours, including TNBC, highlighted drug resistance as the major reason for limiting TKIs efficacy in cancer therapy and point to the requirement of studies aimed to clarify the responsible molecular mechanisms [31].

Here, we first identify a link between aberrant RTKs signalling and NRF2 in TNBC and then we demonstrate that its targeting ameliorate the therapeutic response to standard chemotherapy. We focused our attention on MET, which is overexpressed in about 40% of BC patients and in more than 50% of TNBC [47]. MET overexpression correlates with tumour progression and aggressiveness [48]. Using murine and human TNBC cellular models, we demonstrated that MET sustains NRF2 expression, nuclear localization, and transcriptional activity. NRF2 regulates more than 200 target genes [7] and here we showed that MET inhibition decreased NRF2 transcriptional activity, exemplified by the downregulation of the expression of some canonical targets, like *GCLC*, *MAFG*,* SLC7A11* and *GSR*. Furthermore, transcriptomic analysis identified NRF2 among those TFs whose activity is significantly decreased upon MET blockage.

It has been shown that MET-NRF2-HO1 axis has a fundamental role in reducing oxidative stress in renal cancer [49, 50], suggesting that it would be valuable to investigate whether a similar interdependence exists in TNBC. The idea of NRF2 targeting to overcome cancer cell resistance to therapy is well supported by the literature [39]. Here, we showed that treatment with NRF2 specific inhibitor ML-385 strongly affected human TNBC cellular clonogenicity potential, thus uncovering NRF2 as relevant therapeutic target. NRF2 blockage had no effects on the non-tumorigenic human mammary cell line MCF10-A, highlighting its specificity in cancer cells.

It is well known that standard chemotherapeutic agents such as PTX strongly cause ROS accumulation leading to NRF2 activation, likely responsible for cell protection to chemotherapy [36, 51]. It has been shown that NRF2 targeting with naturally derived extracts increases sensitivity to PTX in vitro and in vivo prostate cancer models [52]. Here, we demonstrate that ML-385 significantly ameliorates cell sensitivity to PTX treatment in TNBC cellular models as well as in PDOs, strengthening the relevance also in relation to TNBC heterogeneity. Our study supports the repositioning of MET and SRC inhibitors as a valuable strategy to hit NRF2 signalling and therefore sensitize cancer cells to PTX treatment. Mechanistically, both NRF2 and MET blockage enhanced ROS accumulation and DNA damage, enhancing TNBC responsiveness to PTX. The relevance of this mechanism is strengthened by a positive correlation that we showed between levels of several RTKs and NRF2, and particularly between high levels of EGFR and NRF2 and a worse TNBC prognosis. EGFR is often deregulated in TNBC and several EGFR inhibitors have been shown to ameliorate the therapeutic response [53]. In this regard, the co-targeting of both MET and EGFR could represent another valuable strategy to improve NRF2 targeting and counteract TNBC [47, 54].

By investigating the molecular mechanism linking MET to NRF2, we provide evidence for a key role of SRC kinase. SRC is an important downstream mediator of several RTKs including MET [55]. It is often upregulated in TNBC and its activation can cause the phosphorylation of several downstream substrates including transcription factors [37]. Here, we also demonstrated that the pharmacological inhibition of SRC activity, using Dasatinib, reduced NRF2 protein levels, nuclear localization and activity in human TNBC cell lines, similarly to what we previously reported in GBM cells [24]. TNBC patients present high levels of *SRC* expression and a simultaneous high levels of *SRC* and *NRF2* correlate with worse clinical outcomes. Moreover, SRC inhibition sensitize PDO to PTX, recapitulating the effects of NRF2 or MET blockage. We have previously shown that in GBM cells SRC can sustain p62 phosphorylation on S349, therefore promoting p62-KEAP1 interaction allowing NRF2 protein stabilization [24]. Here we provide evidence for a conserved mechanism in TNBC cells, through which aberrant activation of MET sustains SRC activity, promoting p62-KEAP1 interaction and upregulation of NRF2 expression and signalling. Future experiments will clarify whether other RTKs are equally capable to impinge on NRF2 in a SRC dependent or independent manner. Moreover, it will be interesting to extend our studies to other TKIs, to uncover more effective compounds and/or other RTKs that may represent valuable targets to modulate NRF2 and therefore be exploited to ameliorate TNBC sensitivity to standard therapeutic approaches. We are aware that our findings are based on preclinical models and bioinformatic analyses on patient datasets. Future studies by performing immunohistochemical analyses on patient samples and by assessing the effectiveness of combinatorial treatments in TNBC in vivo models will be necessary to further strengthen the significance of our discovery.

## Conclusions

Our work highlights the existence of a novel functional interplay between MET and NRF2 in TNBC. The therapeutic relevance is demonstrated by the effectiveness of its targeting to confer responsiveness of TNBC models to PTX, offering a novel therapeutic option and putative signature for TNBC.

## Supplementary Information

Supplementary Material 1. Figure S1 A) Expression levels of *AXL, TGFBR1 *and *PDGFRA* across the four different BC subtypes from the TCGA dataset. B) Kaplan–Meier curves show the probability of overall survival of non-TNBC patients with different expression levels of* MET/NRF2* (left) and *EGFR/NRF2 *(right). Statistical analysis: A) One-way ANOVA followed by Tukey’s multiple comparison statistical test was performed. B) Survival data derived from TCGA dataset. ns: not significant. **** p<0.0001.

Supplementary Material 2. Figure S2 A) Immunoblotting analysis of pY_1068_EGFR, EGFR, pY_1234/35_MET and MET in MGT-13 cells after 16 h of PHA treatment. Actin was used as a loading control. Immunoblotting (B) and relative densitometric analyses (C) of MET, NRF2, HO-1 and p62 in MGT-13 cells transiently silenced for MET expression (siMET). Actin was used as a loading control. D) Immunofluorescence (left) and relative quantification analysis (right) of NRF2 (red) nuclear intensity in MGT-13 cells transiently silenced for MET expression (siMET). DNA (Hoechst, blue). E) RT-qPCR of NRF2 target genes in MGT-13 cells transiently silenced for MET expression (siMET). Actin was used as housekeeping gene. F) Immunoblotting analysis of pY_1234/35_MET and MET in MGT-13 cells after 24 h of PHA treatment. Actin was used as a loading control. G) PCA based on RNA-seq data for PHA and CTRL in MGT-13 cells. H) Volcano plot. Dots in blue are downregulated genes upon PHA treatment (log_2_Fold Change < -0.7 and p-value <0.05), upregulated are in red (log_2_ Fold Change > 0.7 and p-value <0.05). Barplots showing the most affected pathways by the downregulated (I) and upregulated (J) genes found in the PHA treated samples compared to control. Colouring scheme according to the Enrichment FDR (False Discovery Rate) values. Data from ShinyGO. K) Differential expression of *NFE2L2* compared to CTRL in MGT-13 cells. L) Volcano plot displaying gene expression log_2_ fold change and adjusted p-value of control *vs* siNRF2 conditions. Significant genes, (defined as genes with log_2_ fold change < -0.7 or > 0.7 and p-value <0.05) as blue (downregulated in siNRF2) and red (upregulated) dots. M) Gene ontologies for the downregulated genes upon NRF2 interference. For each term, the fold enrichment is shown on the x axis. Size and colour of the dot indicate the number of downregulated genes contributing to that pathway and the % on the total genes belonging to that pathway, respectively. N) Heatmap showing the comparison between z-scores for genes downregulated in the NRF2 pathway (according to IPA) for control vs PHA and control vs siNRF2 (3 samples for each condition, see x axis). Positive values of z-score (red) indicate upregulation within the sample, negative values (blue) downregulation. Results represent the mean of at least three independent experiments (± SEM or ± SD). Statistical analysis: C-E) Multiple *t*-test. D) Mann-Whitney test according to the normal distribution. PHA: PHA-665752 1 µM, MET inhibitor. * p<0.05; ** p<0.01; *** p<0.001; **** p<0.0001.

Supplementary Material 3. Figure S3 A) Immunoblotting (left) of pY_1234/35_MET, MET and NRF2 in GTL-16 cell line upon 16 h of PHA treatment and relative densitometric analysis (right) of NRF2 protein levels. GAPDH was used as a loading control. B) RT-qPCR of NRF2 target genes in GTL-16 cells after 16 h of PHA treatment. 18S was used as housekeeping gene. C) Immunoblotting of pY_1234/35_MET and MET in MDA-MB-231 and BT-549 cell lines upon 16 h of PHA treatment. Actin was used as loading control. Immunoblotting (D) and relative densitometric analyses (E) of MET, NRF2, HO-1 and p62 in BT-549 cells transiently silencing for MET expression (siMET). Vinculin was used as a loading control. F) Immunofluorescence (left) and relative quantification analysis (right) of NRF2 (red) nuclear intensity in BT-549 cells transiently silenced for MET expression (siMET). DNA (Hoechst, blue). G) RT-qPCR of NRF2 target genes in BT-549 cells transiently silenced for MET expression (siMET). Actin was used as housekeeping gene. H) Immunofluorescence (left) and relative quantification analysis (right) of NRF2 (red) nuclear intensity in human TNBC cell lines after 4 h of serum-free media and 10 minutes of HGF stimulation (50ng/mL). DNA (Hoechst, blue). Results represent the mean of at least three independent experiments (± SEM or ± SD). Statistical analysis: A) Unpaired *t*-test. B-E-G) Multiple *t*-test. F-H) Mann-Whitney test according to the normal distribution. PHA: PHA-665752 1 µM, MET inhibitor. * p<0.05; ** p<0.01; **** p<0.0001.

Supplementary Material 4. Figure S4 Clonogenic assays on BT-549 cells exposed to Paclitaxel in combination with PHA (A) or ML-385 (B) and treated similarly as in Fig.4B. PHA: PHA-665752, MET inhibitor. ML-385: NRF2 inhibitor

Supplementary Material 5. Figure S5 Immunoblotting (A) and relative densitometric analyses (B) of NRF2, p62 and HO-1 in MGT-13 and BT-549 cells upon 24 h of PTX treatment. C-D) RT-qPCR of NRF2 target genes in MGT-13 and BT-549 cells upon 24 h of PTX treatment. E) Immunofluorescence (left) and relative quantification analysis (right) of NRF2 (red) nuclear intensity in MGT-13 and BT-549 cells upon 24 h of PTX treatment. DNA (Hoechst, blue). F) Confocal microscopy analysis (left) and relative quantification (right) of 8-oxo-DG (red) foci/cell in BT-549 cells treated alone or in combination with PTX and PHA. DNA (Hoechst, blue). G) Confocal microscopy analysis (left) and relative quantification (right) of γH2AX (green) positive cells in BT-549 cells treated alone or in combination with PTX and PHA. DNA (Hoechst, blue). Results represent the mean of at least three independent experiments (± SEM or ± SD). Statistical analysis: B-F-G) One-way ANOVA statistical test. C-D) Multiple *t*-test. E) Mann-Whitney test according to the normal distribution. PHA: PHA-665752 3 µM, MET inhibitor. PTX: Paclitaxel 3nM or 30 nM. ns: not significant; * p<0.05; ** p<0.01; **** p<0.0001.

Supplementary Material 6. Figure S6 Immunoblotting of MET, pY_416_SRC and SRC (top) and relative densitometric analyses (bottom) of pY_416_SRC normalized on total SRC in TNBC cell lines transiently silenced for MET expression (siMET). Vinculin was used as loading control. Immunoblotting pY_416_SRC, SRC and NRF2 (B) and relative densitometric analysis of NRF2 (C) in MGT-13 cells transiently transfected with empty vector (pSGT), active SRC (SRC^Y527F^), catalytically inactive SRC (SRC^K295M^). Actin was used as loading control. Immunoblotting (D) and relative densitometric analyses (E) of KEAP1, pS_349_p62 and p62 in MGT-13 cells after 16 h of PHA treatment. Actin was used as loading control. F) Confocal microscopy analyses and relative quantification of co-localizing dots of KEAP1 (red) and p62 (green) in MGT-13 cells upon 16 h of PHA treatment. DNA (Hoechst, blue). 3X digital magnification showing merged signals. Results represent the mean of at least three independent experiments (± SEM or ± SD). Statistical analysis: A-E) Multiple *t*-test. C) One-way ANOVA statistical test. F) Mann-Whitney test according to the normal distribution. PHA: PHA-665752 1 µM, MET inhibitor. * p<0.05; ** p<0.01; *** p<0.001;

Supplementary Material 7. Figure S7 A) Immunoblotting (top) of NRF2, pY_416_SRC, SRC and relative densitometric analysis (bottom) of NRF2 protein levels in BT-549 cells upon 16 h of DAS treatment. Actin was used as a loading control. B) Immunofluorescence (left) and relative quantification (right) analysis of NRF2 (red) nuclear intensity in BT-549 cells upon 16 h of DAS treatment. DNA (Hoechst, blue). Results represent the mean of at least three independent experiments (± SEM). Statistical analysis: A) Unpaired *t*-test. B) Mann-Whitney test according to the normal distribution. DAS: Dasatinib 50 nM; SRC inhibitor.* p<0.05.

Supplementary Material 8. Figure S8 A) Dose-response curves for PTX, PHA, DAS, ML-385 tested in PDO-21, PDO-43 and PDO-46. B) Table representing the calculation of the IC50 for each treatment in all PDOs. 4X digital magnification of bright-field images of organoids size of PDO-43 (C) and PDO-46 (D) cultures treated with PHA, DAS, ML-385 combined with PTX. Scale bar: 400 µm. E) Percentage of small, medium and large PDOs treated as in (C-D). F) Table representing the staining of MET and NRF2 in the 15 TNBC biopsies analysed by IHC. G) Representative examples of immunohistochemical staining of NRF2 and MET (20x). A and B: Case with negative MET and weak NRF2 expression. C and D: Case with moderate-to-weak MET staining and strong NRF2 expression. Results represent the mean of at least three independent experiments (± SEM). Statistical analysis: E) Two-way ANOVA followed by Tukey’s multiple comparison test (* respect to CTR, $ respect to inhibitors and # respect to PTX). PHA: PHA-665752, MET inhibitor. DAS: Dasatinib. ML-385: NRF2 inhibitor. PTX: Paclitaxel. ns: not significant; *, $, # p<0.05; **, $$, ## p<0.01; ***, $$$, ### p<0.001; ****, $$$$, #### p<0.0001.

## Data Availability

The dataset generated and analysed during the current study [https://urldefense.com/v3/\_\_https://www.ncbi.nlm.nih.gov/geo/query/acc.cgi?acc=GSE290518\_\_;;!!O5Bi4QcV!DnSpwUVkhFP9m6fIVSd0lrfHzAHaOr_b0NUxodpYyxw4-IZ3XNhAcreWudH_GAaWXt8-zaiY8_Ty7E4D8PbGz8KKaw$](https:/urldefense.com/v3/__https:/www.ncbi.nlm.nih.gov/geo/query/acc.cgi?acc=GSE290518__;!!O5Bi4QcV!DnSpwUVkhFP9m6fIVSd0lrfHzAHaOr_b0NUxodpYyxw4-IZ3XNhAcreWudH_GAaWXt8-zaiY8_Ty7E4D8PbGz8KKaw$) is not publicly available before publication, but **reviewers can gain access to the GEO dataset using the following token: uvcjuymsnxibtwt.**.

## Abbreviations

BC: Breast Cancer
TNBC: Triple Negative Breast Cancer
NRF2: Nuclear Factor Erythroid 2-related factor 2
PTKs: Protein Tyrosine Kinases
TKIs: Tyrosine Kinase Inhibitors
ER: Estrogen Receptor
PR: Progesterone Receptor
HER2: Human Epidermal Growth Factor Receptor 2
KEAP1: Kelch-like ECH-associated protein 1
OS: Overall Survival
DFS: Disease Free Survival
RTKs: Receptor Tyrosine Kinanes
nRTKs: Non-Receptor Tyrosine Kinases
PDOs: Patient-Derived Organoids
MGT: Mammary Gland Tumour
TFs: Transcription Factors
PTX: Paclitaxel
DAS: Dasatinib
PHA: PHA-665752
