# Supplementary figures and images for: Targeting of a novel interplay between MET tyrosine kinase and NRF2 enhances sensitivity to Paclitaxel in triple negative breast cancer
Source: J Exp Clin Cancer Res. 2026 Jan 20;45:52. doi: 10.1186/s13046-025-03625-y (PMC12903676; doi:10.1186/s13046-025-03625-y)

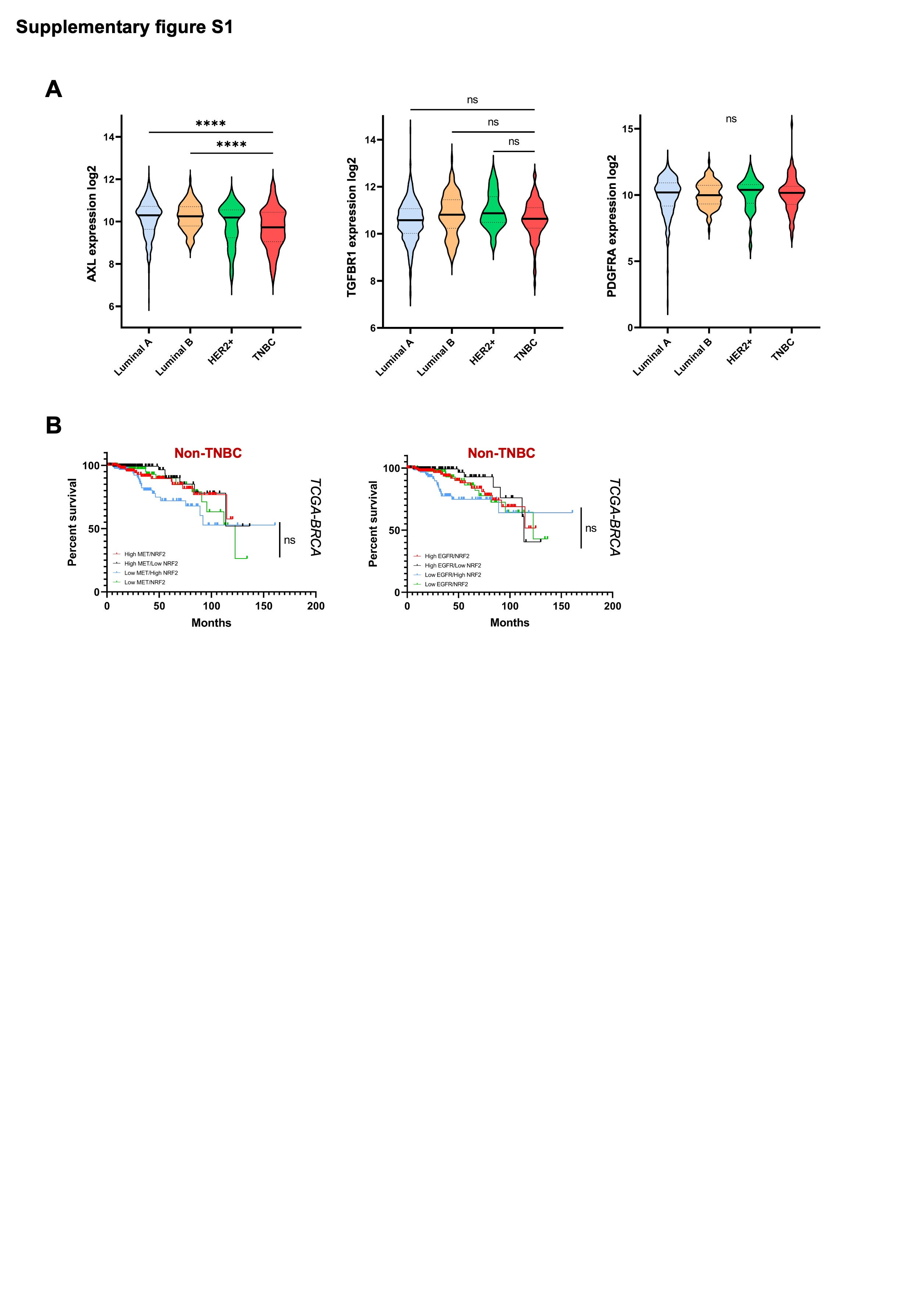

Supplement: Supplementary file 1 — Supplementary Material 1. Figure S1 A) Expression levels of AXL, TGFBR1 and PDGFRA across the four different BC subtypes from the TCGA dataset. B) Kaplan–Meier curves show the probability of overall survival of non-TNBC patients with different expression levels of MET/NRF2 (left) and EGFR/NRF2 (right). Statistical analysis: A) One-way ANOVA followed by Tukey’s multiple comparison statistical test was performed. B) Survival data derived from TCGA dataset. ns: not significant. **** p<0.0001. [file 13046_2025_3625_MOESM1_ESM.tiff]

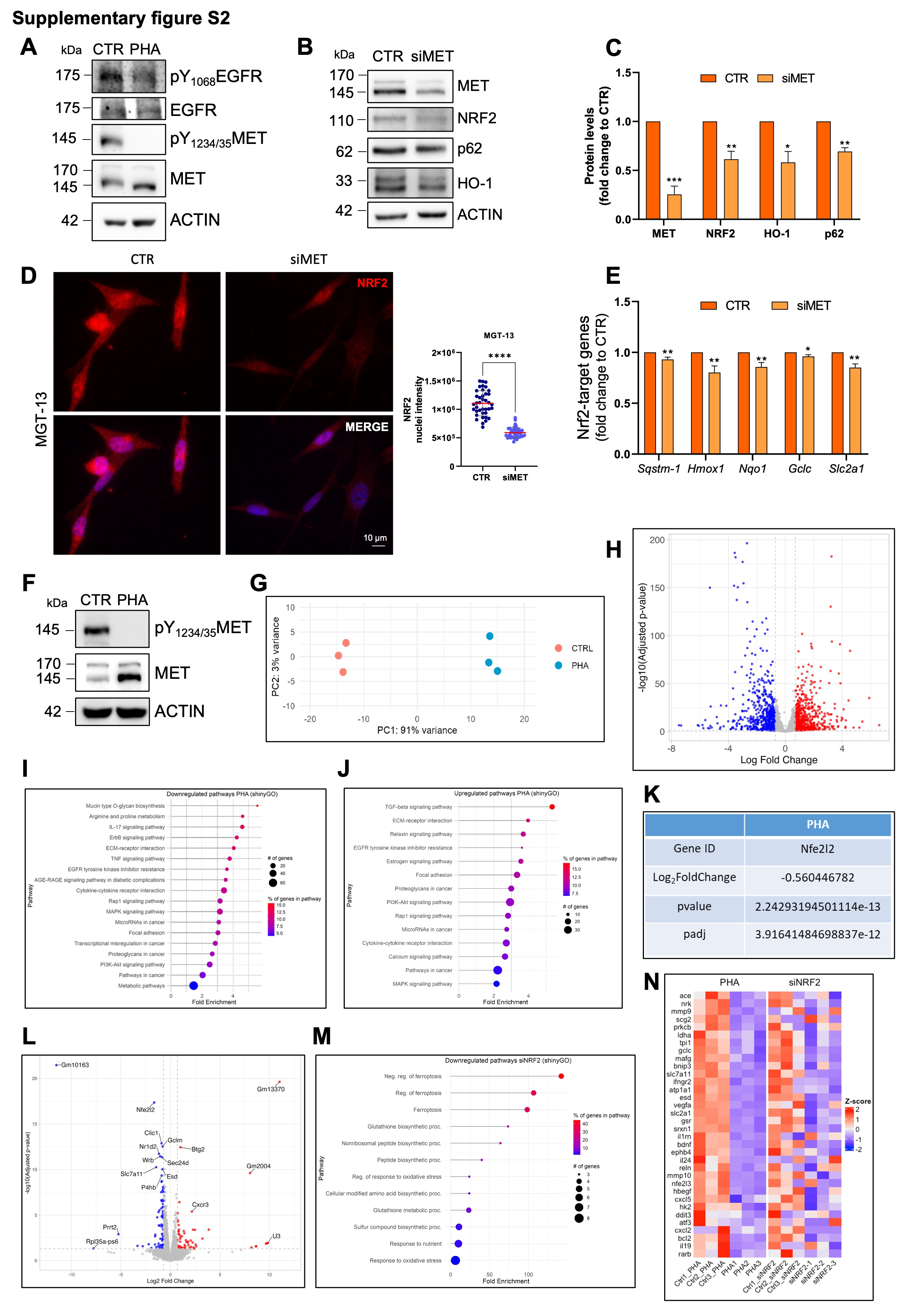

Supplement: Supplementary file 2 — Supplementary Material 2. Figure S2 A) Immunoblotting analysis of pY1068EGFR, EGFR, pY1234/35MET and MET in MGT-13 cells after 16 h of PHA treatment. Actin was used as a loading control. Immunoblotting (B) and relative densitometric analyses (C) of MET, NRF2, HO-1 and p62 in MGT-13 cells transiently silenced for MET expression (siMET). Actin was used as a loading control. D) Immunofluorescence (left) and relative quantification analysis (right) of NRF2 (red) nuclear intensity in MGT-13 cells transiently silenced for MET expression (siMET). DNA (Hoechst, blue). E) RT-qPCR of NRF2 target genes in MGT-13 cells transiently silenced for MET expression (siMET). Actin was used as housekeeping gene. F) Immunoblotting analysis of pY1234/35MET and MET in MGT-13 cells after 24 h of PHA treatment. Actin was used as a loading control. G) PCA based on RNA-seq data for PHA and CTRL in MGT-13 cells. H) Volcano plot. Dots in blue are downregulated genes upon PHA treatment (log2Fold Change < -0.7 and p-value <0.05), upregulated are in red (log2 Fold Change > 0.7 and p-value <0.05). Barplots showing the most affected pathways by the downregulated (I) and upregulated (J) genes found in the PHA treated samples compared to control. Colouring scheme according to the Enrichment FDR (False Discovery Rate) values. Data from ShinyGO. K) Differential expression of NFE2L2 compared to CTRL in MGT-13 cells. L) Volcano plot displaying gene expression log2 fold change and adjusted p-value of control vs siNRF2 conditions. Significant genes, (defined as genes with log2 fold change < -0.7 or > 0.7 and p-value <0.05) as blue (downregulated in siNRF2) and red (upregulated) dots. M) Gene ontologies for the downregulated genes upon NRF2 interference. For each term, the fold enrichment is shown on the x axis. Size and colour of the dot indicate the number of downregulated genes contributing to that pathway and the % on the total genes belonging to that pathway, respectively. N) Heatmap showing the comparis [file 13046_2025_3625_MOESM2_ESM.tiff]

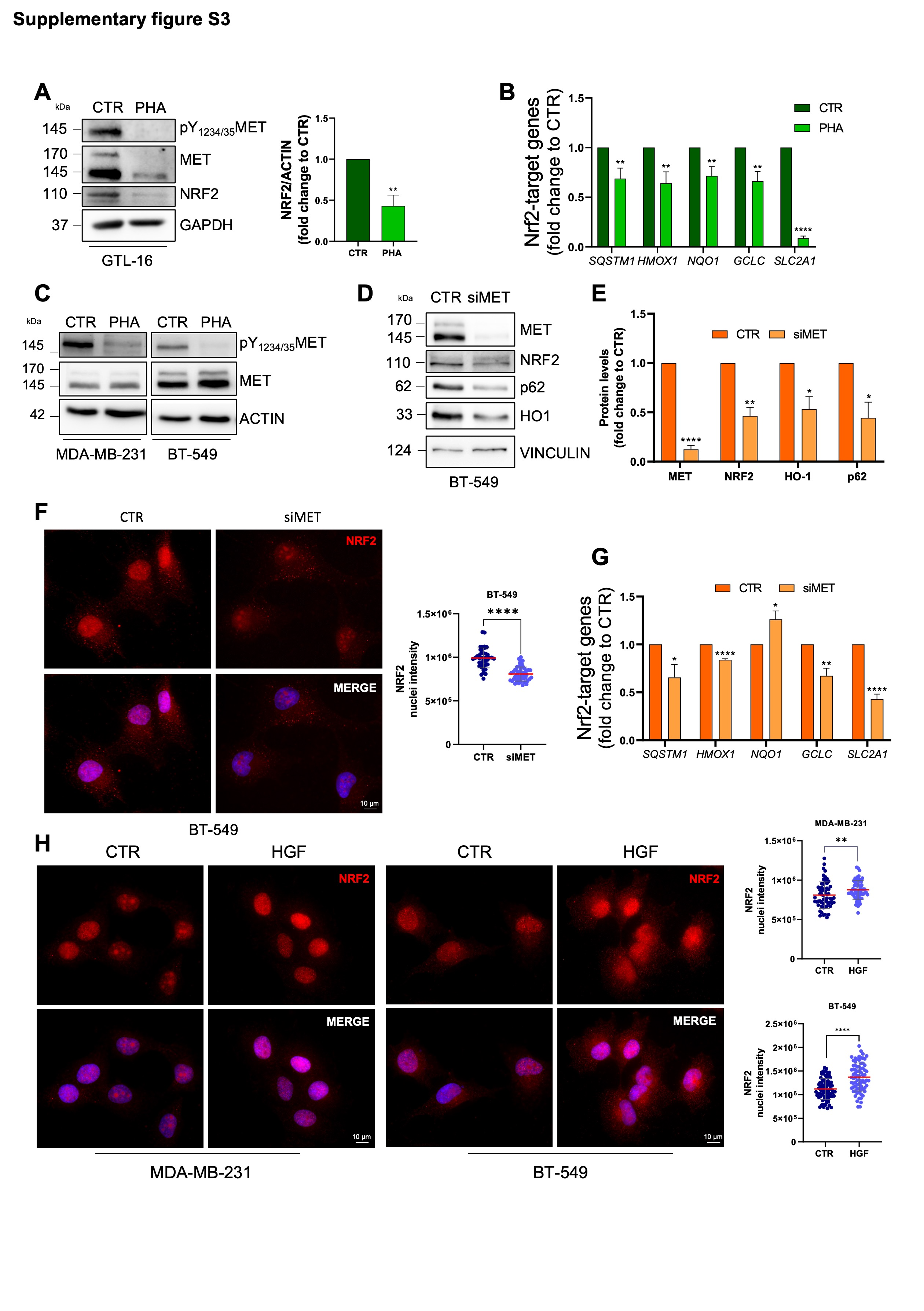

Supplement: Supplementary file 3 — Supplementary Material 3. Figure S3 A) Immunoblotting (left) of pY1234/35MET, MET and NRF2 in GTL-16 cell line upon 16 h of PHA treatment and relative densitometric analysis (right) of NRF2 protein levels. GAPDH was used as a loading control. B) RT-qPCR of NRF2 target genes in GTL-16 cells after 16 h of PHA treatment. 18S was used as housekeeping gene. C) Immunoblotting of pY1234/35MET and MET in MDA-MB-231 and BT-549 cell lines upon 16 h of PHA treatment. Actin was used as loading control. Immunoblotting (D) and relative densitometric analyses (E) of MET, NRF2, HO-1 and p62 in BT-549 cells transiently silencing for MET expression (siMET). Vinculin was used as a loading control. F) Immunofluorescence (left) and relative quantification analysis (right) of NRF2 (red) nuclear intensity in BT-549 cells transiently silenced for MET expression (siMET). DNA (Hoechst, blue). G) RT-qPCR of NRF2 target genes in BT-549 cells transiently silenced for MET expression (siMET). Actin was used as housekeeping gene. H) Immunofluorescence (left) and relative quantification analysis (right) of NRF2 (red) nuclear intensity in human TNBC cell lines after 4 h of serum-free media and 10 minutes of HGF stimulation (50ng/mL). DNA (Hoechst, blue). Results represent the mean of at least three independent experiments (± SEM or ± SD). Statistical analysis: A) Unpaired t-test. B-E-G) Multiple t-test. F-H) Mann-Whitney test according to the normal distribution. PHA: PHA-665752 1 µM, MET inhibitor. * p<0.05; ** p<0.01; **** p<0.0001. [file 13046_2025_3625_MOESM3_ESM.tiff]

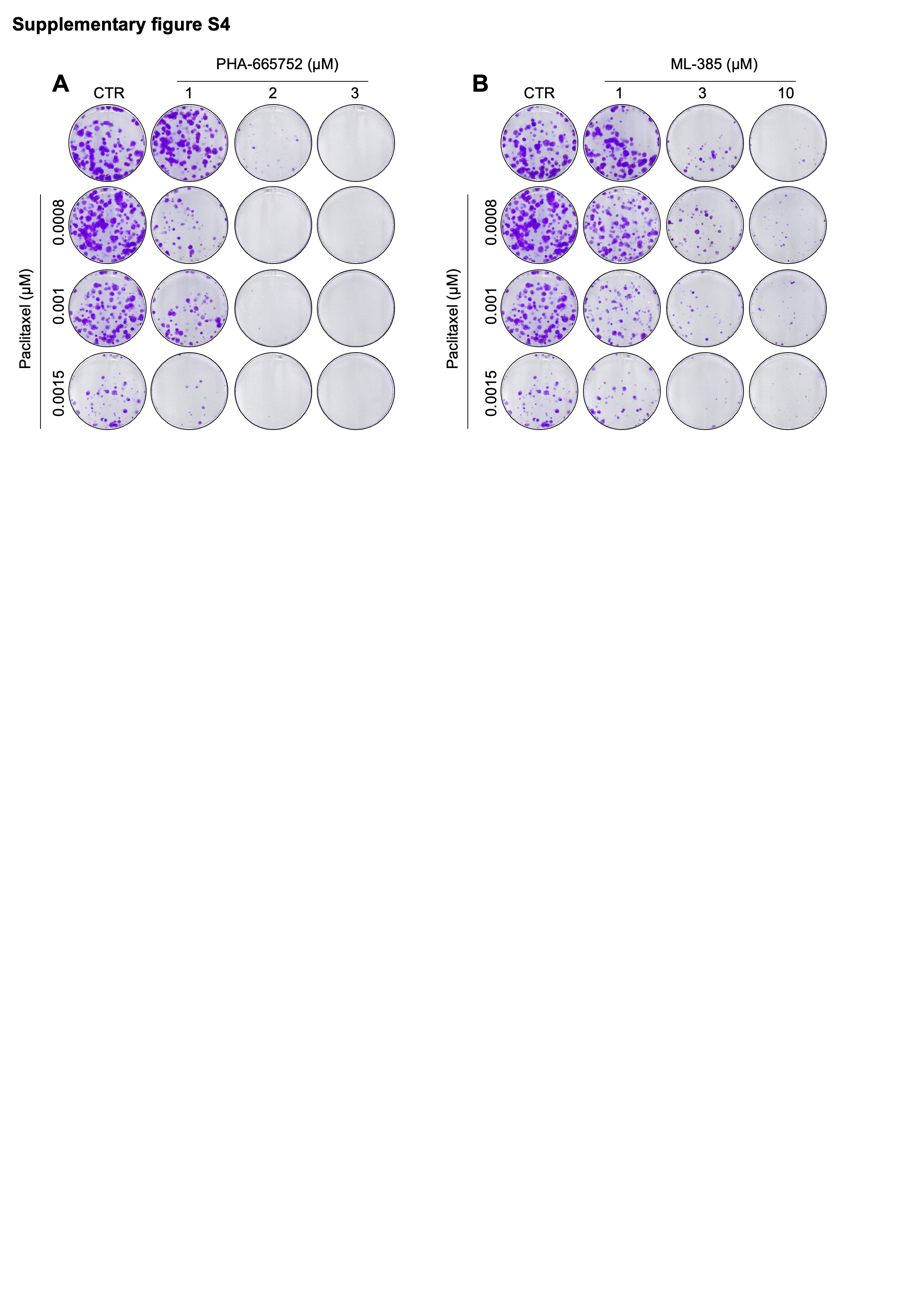

Supplement: Supplementary file 4 — Supplementary Material 4. Figure S4 Clonogenic assays on BT-549 cells exposed to Paclitaxel in combination with PHA (A) or ML-385 (B) and treated similarly as in Fig.4B. PHA: PHA-665752, MET inhibitor. ML-385: NRF2 inhibitor [file 13046_2025_3625_MOESM4_ESM.tiff]

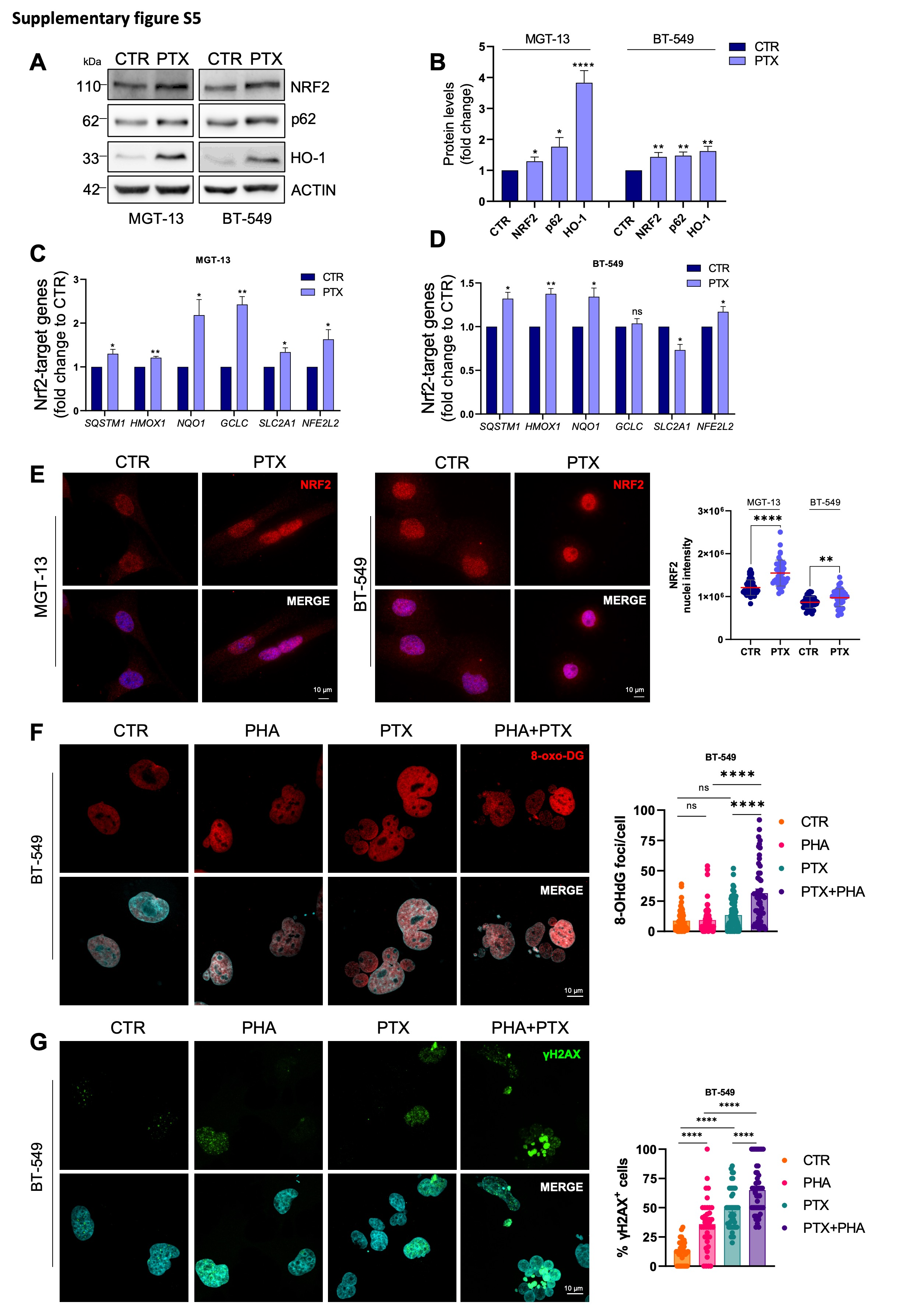

Supplement: Supplementary file 5 — Supplementary Material 5. Figure S5 Immunoblotting (A) and relative densitometric analyses (B) of NRF2, p62 and HO-1 in MGT-13 and BT-549 cells upon 24 h of PTX treatment. C-D) RT-qPCR of NRF2 target genes in MGT-13 and BT-549 cells upon 24 h of PTX treatment. E) Immunofluorescence (left) and relative quantification analysis (right) of NRF2 (red) nuclear intensity in MGT-13 and BT-549 cells upon 24 h of PTX treatment. DNA (Hoechst, blue). F) Confocal microscopy analysis (left) and relative quantification (right) of 8-oxo-DG (red) foci/cell in BT-549 cells treated alone or in combination with PTX and PHA. DNA (Hoechst, blue). G) Confocal microscopy analysis (left) and relative quantification (right) of γH2AX (green) positive cells in BT-549 cells treated alone or in combination with PTX and PHA. DNA (Hoechst, blue). Results represent the mean of at least three independent experiments (± SEM or ± SD). Statistical analysis: B-F-G) One-way ANOVA statistical test. C-D) Multiple t-test. E) Mann-Whitney test according to the normal distribution. PHA: PHA-665752 3 µM, MET inhibitor. PTX: Paclitaxel 3nM or 30 nM. ns: not significant; * p<0.05; ** p<0.01; **** p<0.0001. [file 13046_2025_3625_MOESM5_ESM.tiff]

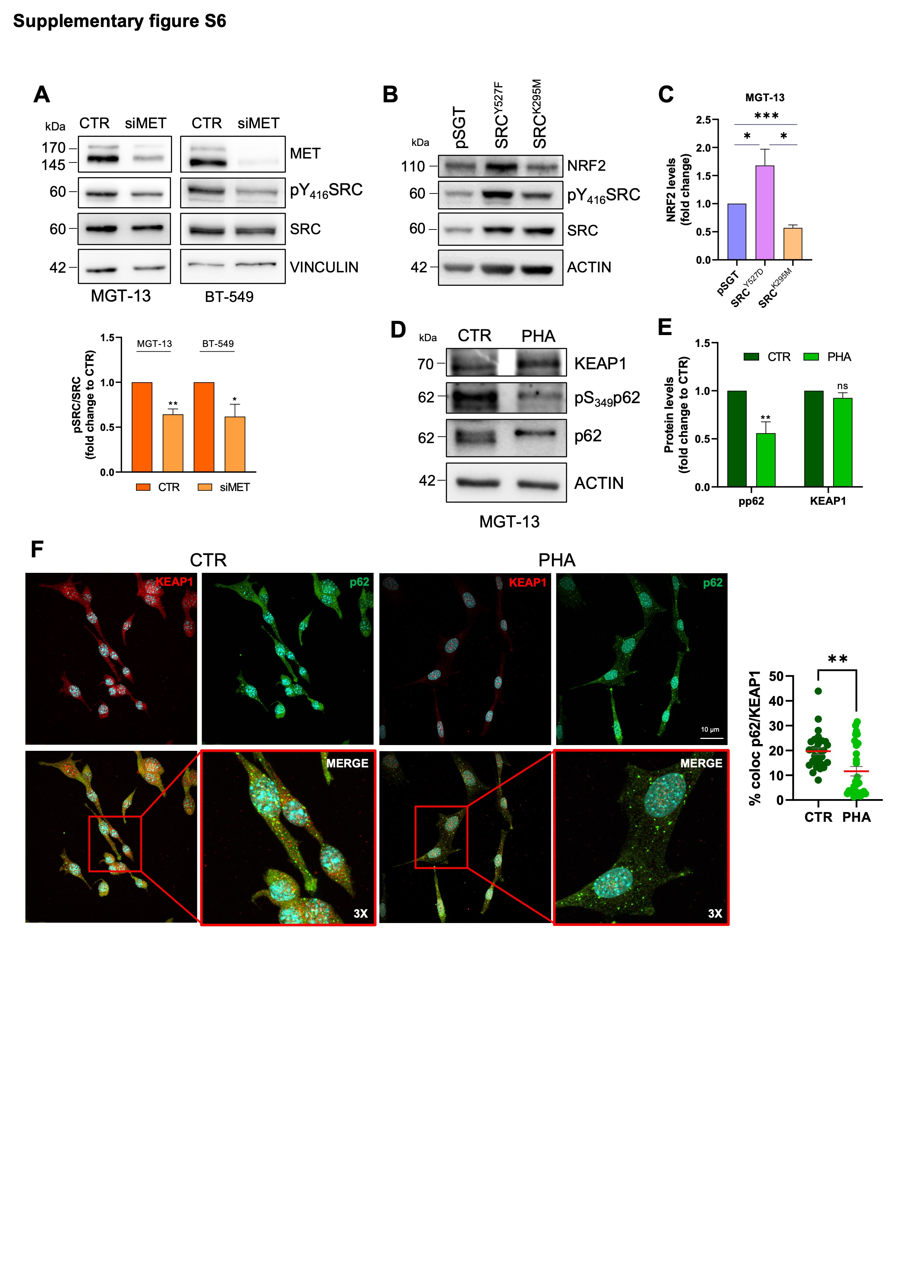

Supplement: Supplementary file 6 — Supplementary Material 6. Figure S6 Immunoblotting of MET, pY416SRC and SRC (top) and relative densitometric analyses (bottom) of pY416SRC normalized on total SRC in TNBC cell lines transiently silenced for MET expression (siMET). Vinculin was used as loading control. Immunoblotting pY416SRC, SRC and NRF2 (B) and relative densitometric analysis of NRF2 (C) in MGT-13 cells transiently transfected with empty vector (pSGT), active SRC (SRCY527F), catalytically inactive SRC (SRCK295M). Actin was used as loading control. Immunoblotting (D) and relative densitometric analyses (E) of KEAP1, pS349p62 and p62 in MGT-13 cells after 16 h of PHA treatment. Actin was used as loading control. F) Confocal microscopy analyses and relative quantification of co-localizing dots of KEAP1 (red) and p62 (green) in MGT-13 cells upon 16 h of PHA treatment. DNA (Hoechst, blue). 3X digital magnification showing merged signals. Results represent the mean of at least three independent experiments (± SEM or ± SD). Statistical analysis: A-E) Multiple t-test. C) One-way ANOVA statistical test. F) Mann-Whitney test according to the normal distribution. PHA: PHA-665752 1 µM, MET inhibitor. * p<0.05; ** p<0.01; *** p<0.001; [file 13046_2025_3625_MOESM6_ESM.tiff]

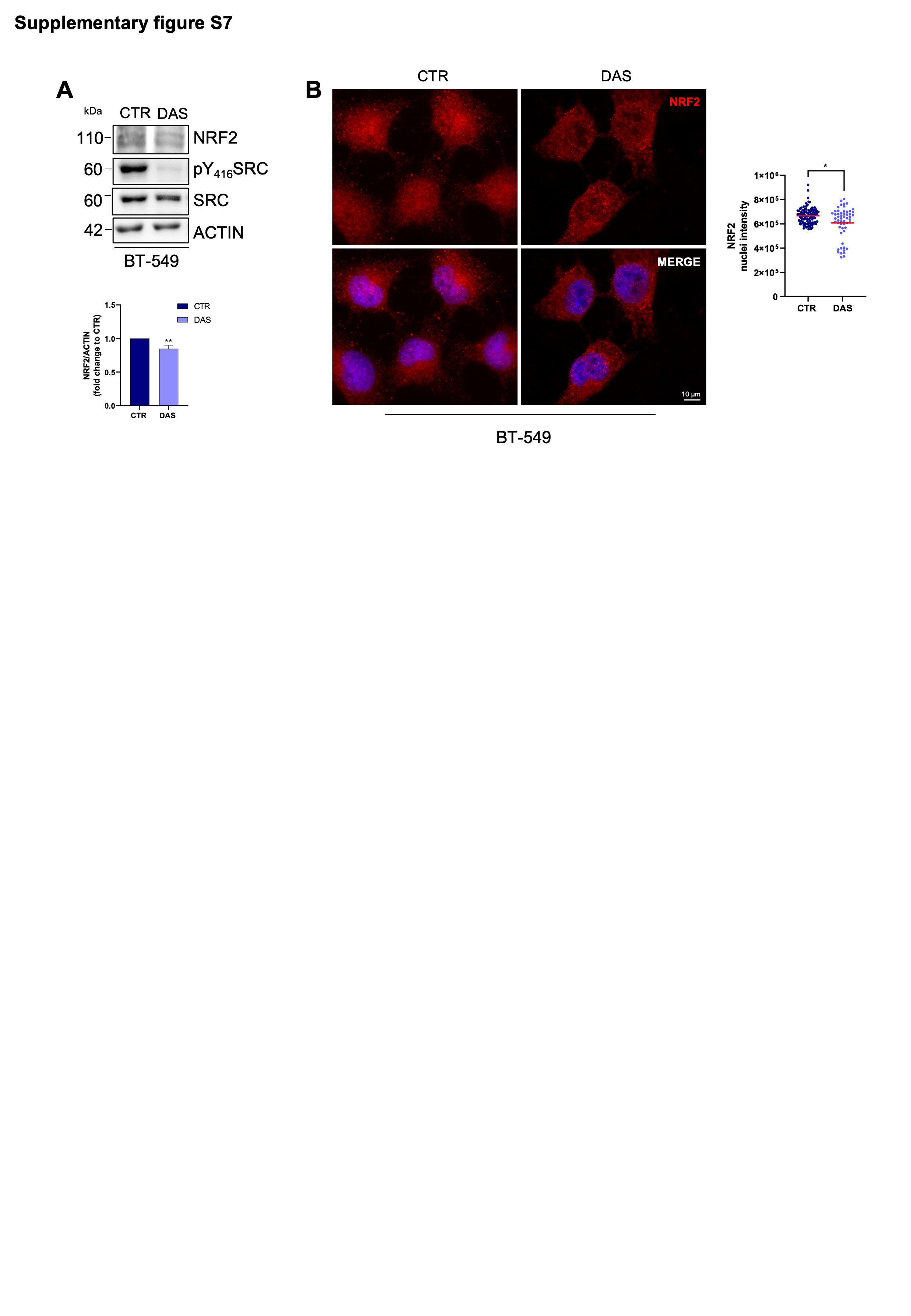

Supplement: Supplementary file 7 — Supplementary Material 7. Figure S7 A) Immunoblotting (top) of NRF2, pY416SRC, SRC and relative densitometric analysis (bottom) of NRF2 protein levels in BT-549 cells upon 16 h of DAS treatment. Actin was used as a loading control. B) Immunofluorescence (left) and relative quantification (right) analysis of NRF2 (red) nuclear intensity in BT-549 cells upon 16 h of DAS treatment. DNA (Hoechst, blue). Results represent the mean of at least three independent experiments (± SEM). Statistical analysis: A) Unpaired t-test. B) Mann-Whitney test according to the normal distribution. DAS: Dasatinib 50 nM; SRC inhibitor.* p<0.05. [file 13046_2025_3625_MOESM7_ESM.tiff]

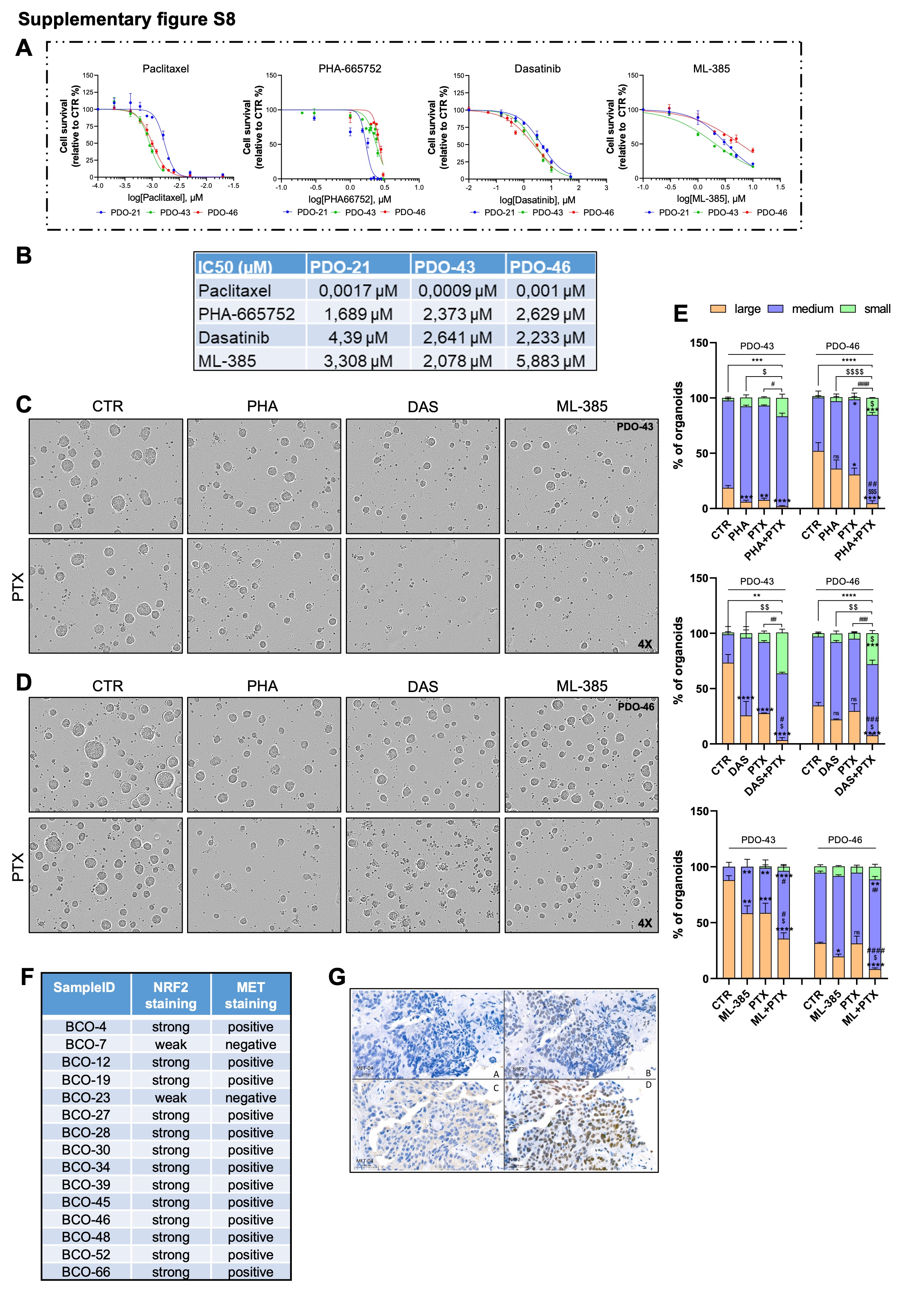

Supplement: Supplementary file 8 — Supplementary Material 8. Figure S8 A) Dose-response curves for PTX, PHA, DAS, ML-385 tested in PDO-21, PDO-43 and PDO-46. B) Table representing the calculation of the IC50 for each treatment in all PDOs. 4X digital magnification of bright-field images of organoids size of PDO-43 (C) and PDO-46 (D) cultures treated with PHA, DAS, ML-385 combined with PTX. Scale bar: 400 µm. E) Percentage of small, medium and large PDOs treated as in (C-D). F) Table representing the staining of MET and NRF2 in the 15 TNBC biopsies analysed by IHC. G) Representative examples of immunohistochemical staining of NRF2 and MET (20x). A and B: Case with negative MET and weak NRF2 expression. C and D: Case with moderate-to-weak MET staining and strong NRF2 expression. Results represent the mean of at least three independent experiments (± SEM). Statistical analysis: E) Two-way ANOVA followed by Tukey’s multiple comparison test (* respect to CTR, $ respect to inhibitors and # respect to PTX). PHA: PHA-665752, MET inhibitor. DAS: Dasatinib. ML-385: NRF2 inhibitor. PTX: Paclitaxel. ns: not significant; *, $, # p<0.05; **, $$, ## p<0.01; ***, $$$, ### p<0.001; ****, $$$$, #### p<0.0001. [file 13046_2025_3625_MOESM8_ESM.tiff]
